# Dysregulated glucuronic acid metabolism exacerbates hepatocellular carcinoma progression and metastasis through the TGFβ signalling pathway

**DOI:** 10.1002/ctm2.995

**Published:** 2022-08-17

**Authors:** Qingzhu Gao, Bin Cheng, Chang Chen, Chong Lei, Xue Lin, Dan Nie, Jingjing Li, Luyi Huang, Xiaosong Li, Kai Wang, Ailong Huang, Ni Tang

**Affiliations:** ^1^ Key Laboratory of Molecular Biology for Infectious Diseases (Ministry of Education), Institute for Viral Hepatitis, Department of Infectious Diseases The Second Affiliated Hospital Chongqing Medical University Chongqing China; ^2^ Institute of Life Sciences Chongqing Medical University Chongqing China; ^3^ Department of Gastroenterology Chongqing Hospital of Traditional Chinese Medicine Chongqing China; ^4^ Clinical Molecular Medicine Testing Center The First Affiliated Hospital of Chongqing Medical University Chongqing China

**Keywords:** hepatocellular carcinoma metastasis, TGFβ/Smad signalling, UDP‐GlcUA, UGDH

## Abstract

**Background:**

Glucuronic acid metabolism participates in cellular detoxification, extracellular matrix remodeling and cell adhesion and migration. Here, we aimed to explore the crosstalk between dysregulated glucuronic acid metabolism and crucial metastatic signalling in glutathione S‐transferase zeta 1 (GSTZ1)‐deficient hepatocellular carcinoma (HCC).

**Methods:**

Transwell, HCC xenograft and *Gstz1* ^−/‐^ mouse models were used to examine the role of GSTZ1 in HCC metastasis. Non‐targeted and targeted metabolomics and global transcriptomic analyses were performed to screen significantly altered metabolic and signalling pathways in GSTZ1 overexpressing hepatoma cells. Further, RNA‐binding protein immunoprecipitation, Biotin‐RNA pull‐down, mRNA decay assays and luciferase reporter assays were used to explore the interaction between RNA and RNA‐binding proteins.

**Results:**

GSTZ1 was universally silenced in both human and murine HCC cells, and its deficiency contributed to HCC metastasis in vitro and in vivo. UDP‐glucose 6‐dehydrogenase (UGDH)‐mediated UDP‐glucuronic acid (UDP‐GlcUA) accumulation promoted hepatoma cell migration upon GSTZ1 loss. UDP‐GlcUA stabilized *TGFβR1* mRNA by enhancing its binding to polypyrimidine tract binding protein 3, contributing to the activation of TGFβ/Smad signalling. UGDH or TGFβR1 blockade impaired HCC metastasis. In addition, UGDH up‐regulation and UDP‐GlcUA accumulation correlated with increased metastatic potential and decreased patient survival in GSTZ1‐deficient HCC.

**Conclusions:**

GSTZ1 deficiency and subsequent up‐regulation of the glucuronic acid metabolic pathway promotes HCC metastasis by increasing the stability of *TGFβR1* mRNA and activating TGFβ/Smad signalling. UGDH and a key metabolite, UDP‐GlcUA, may serve as prognostic markers. Targeting UGDH might be a promising strategy for HCC therapy.

## Graphical abstract

1. UDP‐glucose 6‐dehydrogenase (UGDH)‐mediated UDP‐GlcUA accumulation promotes hepatoma cell migration upon GSTZ1 loss.

2. UDP‐GlcUA stabilized *TGFβR1* mRNA by enhancing its binding to PTBP3, contributing to the activation of TGFβ/Smad signalling.

3. UGDH and a key intermediate metabolite, UDP‐GlcUA are strong predictors of an increased risk of HCC metastasis, and targeting UGDH might be a promising strategy for HCC therapy.

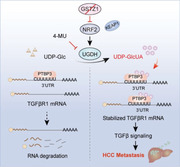

## 1 INTRODUCTION

Tumour metastasis is the major cause of mortality in patients with cancer, including hepatocellular carcinoma (HCC). Several signalling pathways, such as TGFβ, Wnt/β‐catenin, EGF, JAK/STAT, Hippo and HIF, are dysregulated in HCC, leading to uncontrolled cell proliferation and metastasis.^1^, ^2^, ^3^ In addition to the altered signalling and transcriptional networks, recent research has focused on the metabolic plasticity and heterogeneity of metastatic cancer cells.^4^, ^5^ Aberrantly accumulated metabolites, generated from inactivating or activating mutations of metabolic enzymes, such as α‐ketoglutarate, fumarate, and succinate beyond their biosynthetic role, can act as signalling molecules to induce epigenetic deregulation,^6^ promote epithelial–mesenchymal transition (EMT)^7^, ^8^, ^9^ and reinforce the metastatic cascade. The liver is a major metabolic regulator, and HCC onset and progression are frequently accompanied by metabolic rewiring.^10^ However, the interplay between dysregulated cellular metabolism and crucial metastatic signalling in HCC metastasis remains to be fully elucidated.

The glucuronic acid pathway, predominantly found in the cytosol of the liver, is an alternative oxidative pathway for glucose metabolism.^11^ UDP‐glucose 6‐dehydrogenase (UGDH), a key rate‐limiting enzyme in the glucuronic acid pathway, converts UDP‐glucose (UDP‐Glc) to UDP‐glucuronic acid (UDP‐GlcUA), thereby participating in the glucuronidation of endo‐ and xenobiotics and biosynthesis of extracellular matrix component glycosaminoglycans (GAG) such as hyaluronic acid, which is associated with enhanced cell adhesion and migration. Dysregulated glucuronic acid metabolism including increased UGDH expression, altered UDP‐Glc and UDP‐GlcUA levels have been implicated in the growth and metastasis of multiple tumours, such as breast, ovarian and lung cancers.^12^, ^13^, ^14^ Recently, UGDH was identified to have an unexpected role in regulating mRNA stability by enhancing the binding of HuR to *SNAI1* mRNA by converting UDP‐Glc to UDP‐GlcUA. This allows SNAI1 to be stabilized, facilitating EMT, which promotes metastasis.^14^ However, whether and how glucuronic acid metabolic alterations contribute to HCC metastasis remains largely unknown.

Glutathione S‐transferase zeta 1 (GSTZ1) is the penultimate enzyme of tyrosine catabolism and is mainly expressed in the liver and kidney. The major physiological role of GSTZ1 is to catalyze the isomerization of maleylacetoacetate to fumarylacetoacetate.^15^ In addition, GSTZ1 can catalyze the reaction of glutathione with endo‐ and xenobiotics such as dichloroacetic acid, a carcinogenic contaminant of chlorinated water, which is critical for cellular detoxification.^16^ Aberrant GSTZ1 expression has been reported in HCC and breast cancer.^17^ Our previous studies have shown that GSTZ1 may serve as a tumour suppressor in HCC.^18^, ^19^ GSTZ1‐deficiency promotes HCC proliferation by activating the NRF2/IGF1R axis‐mediated anti‐apoptotic pathway. Here, we uncovered a previously unrecognized mechanism by which GSTZ1 deficiency and subsequent up‐regulation of the glucuronic acid metabolic pathway promotes HCC metastasis by increasing the stability of *TGFβR1* mRNA and activating TGFβ/Smad signalling. UGDH and a key intermediate metabolite, UDP‐GlcUA, may serve as prognostic markers, and targeting UGDH might be a promising strategy for HCC therapy.

## 2 MATERIALS AND METHODS

### 2.1 Cell culture and treatment

Human HCC cell lines SK‐Hep1, SNU449, HepG2 and PLC/PRF/5 were obtained from the American Type Culture Collection (VA, USA); Hep3B, Huh7, HEK293T from the Cell Bank of the Chinese Academy of Sciences (Shanghai, China). Immortalized human hepatocytes (MIHA) was a gift from Dr. Ben C.B. Ko (The Hong Kong Polytechnic University). All cell lines were tested negative for mycoplasma. Serum‐starved hepatoma cells were treated with or without UDP‐GlcUA (0.5–1.0 mM, Sigma‐Aldrich U6751), UDP‐Glc (1–5 mM, Millipore 670120), Transforming growth factor beta 1 (TGFβ1, 10 ng/ml, Novoprotein Scientific Inc) or SB431542(10 μM, Selleckchem) for the indicated time.

### 2.2 Patient samples

The paired HCC and non‐tumourous tissues, serum samples were obtained from the Second Affiliated Hospital of Chongqing Medical University between March 2019 and October 2021. This study was approved by the Institutional Review Board of Chongqing Medical University, and informed consent was obtained from all patients.

### 2.3 mRNA decay assays

Cells were pre‐treated with 5,6‐dichloro‐1‐beta‐ribo‐furanosyl benzimidazole (50 μM) to inhibit Ribonucleic acid (RNA) synthesis, and total RNA was isolated at the indicated times using TRIzol reagent and a high‐purity total RNA Rapid Extraction kit. cDNA was prepared using the PrimeScript RT Reagent Kit (Takara, RR047A) and then subjected to real‐time PCR analysis with specific primers (Table S1). Data were normalized to actin used as the endogenous control.

### 2.4 RNA‐binding protein immunoprecipitation

RNA‐binding protein immunoprecipitation (RIP) assay was performed using the EZ‐MagnaRIP Kit (Millipore, # 17–701). Briefly, nuclei pellets of Huh7 cells were lysed using RIP lysis buffer containing RNase inhibitor (Millipore) or a protease inhibitor cocktail. Thereafter, the RIP lysates were mixed with 50μl protein A/G magnetic beads coated with rabbit anti‐PTBP3 antibody or normal rabbit IgG by rotation at 4°C overnight. The immunoprecipitated RNAs were subsequently converted to cDNA for real‐time PCR analysis. Enrichment in the RIPs was calculated as fold change of signal in the immunoprecipitated sample versus the input RNA samples.

### 2.5 RNA‐sequencing analysis

The method used for transcriptomic profiling was described previously.^18^ Briefly, total RNA from Huh7 cells infected with AdGSTZ1 or AdGFP was isolated using TRIzol reagent (Invitrogen). Thereafter, high‐quality RNA with RNA integrity number (RIN) >8.0 was used to construct the cDNA library. RNA‐seq was performed at the Shanghai Novel Bio‐Pharm Technology Co., Ltd. Gene expression profiles were deposited in the Gene Expression Omnibus database (GSE117822).

### 2.6 Biotin‐PRE1 pull‐down assays

Biotin Pull‐down assays were performed by incubating 0.1 mM biotin‐PRE1(5′‐CUUUUUUUCUUUUUUUCUUUUUUU‐3′) with the Huh7 cell lysates or purified recombinant His‐PTBP3‐ RNA recognition motifs (RRM3/4) (5 μg) for 0.5 h at 25°C. Biotin‐PRE1‐Mut (0.1 mM; 5′‐CAUAUAUACAUAUAUA CAUAUAUA‐3′) served as a negative control. Complexes were pulled down using Pierce streptavidin agarose beads and then immunoblotted with the indicated antibodies (Table S2).

### 2.7 Metabolite detection and analysis

Untargeted metabolomic profiling of GSTZ1‐overexpressing cells was performed using ultra‐high‐performance liquid chromatography (Agilent 1290 Infinity LC; Agilent Technologies, Santa Clara, CA, USA) coupled with quadrupole time‐of‐flight mass spectrometry (UHPLC‐QTOF/MS) via electrospray ionization at Shanghai Applied Protein Technology Co., Ltd, Shanghai, China.^20^ Metabolomics data were deposited in the metabolights database (www.ebi.ac.uk/metabolights/MTBLS4908).

UDP‐Glc and UDP‐GlcUA were quantified using liquid chromatography‐tandem MS analysis. For cell samples, 1 × 10^6^ cells were washed with PBS and quenched with liquid nitrogen, followed by the addition of the 2‐chloro‐D‐phenylalanine internal standard. Afterward, liver tumour tissues (50 mg) were homogenized with an internal standard using a tissue homogenizer (Bionoon Technology Inc., Shanghai, China). Serum samples were then mixed with the internal standard using a vortex mixer for 5 s. Subsequently, cells, tissue homogenates, or serum samples were vigorously mixed with a mixture of methanol and acetonitrile (1:1, *v*:*v*). After incubation on ice for 15 min, the lysates were centrifuged at 12 000 × *g* at 4°C for 10 min, and then 10 μl of the supernatant was injected into an Agilent 1290 Infinity II liquid chromatograph coupled to an Agilent 6495c mass spectrometer (Agilent Technologies). Separation was achieved on a Welch Ultimate HILIC Amphion II column (2.1 × 100 mm, 3 μm; Welch Materials Inc., West Haven, CT, USA) with Ammonium formate solution (5 mM) as mobile phase A, and acetonitrile as mobile phase B. Multiple reaction monitoring (MRM) in negative mode was used for MS analysis. The precursor ion, product ion and collision energy for UDP‐Glc were set as 565.1, 323.0 and 25, and those for UDP‐GlcUA were 579.0, 403.0 and 25, respectively. The concentrations of UDP‐Glc and UDP‐GlcUA in the samples were calculated based on the slope of the calibration curves generated using pooled authentic samples (to mimic the matrix), the 2‐chloro‐D‐phenylalanine internal standard, and the UDP‐GlcUA and UDP‐Glc standards purchased from Sigma‐Aldrich with purities greater than 99%.

### 2.8 Animal models and treatment

Four‐week‐old male BALB/c nude mice were used to construct orthotopic metastatic HCC models via tail‐vein injection. Initially, 2 × 10^6^ Huh7 or SNU‐449 cells suspended in PBS were implanted into the lateral tail veins of each mouse. At 10 weeks post‐implantation, mice were sacrificed, and lung and liver tissues were harvested for histological examination.

For the diethylnitrosamine (DEN) and CCl_4_‐induced mouse model of HCC, C57BL/6J WT and *Gstz1* ^−/−^ mice were given a single intraperitoneal injection of DEN (75 mg/kg) at 2 weeks of age, followed by repeated administration of 10% carbon tetrachloride (CCl_4_) (2 ml/kg) intraperitoneally twice a week for 12 weeks, and received phenobarbital diet at a concentration of 0.06% from week 4 to the final sacrifice^21^ (Table S3). In the *Gstz1* ^−/−^ + sg*Ugdh* group, mice were intravenously infected with sg*Ugdh*‐pSECC or sgControl‐pSECC lentiviruses through the tail vein at 8 weeks of age. In the *Gstz1* ^−/−^ + SB431542 group, mice were intraperitoneally administered SB431542(10 mg/kg/day) or vehicle (*n* = 6 per group) for 8 weeks. The mice were sacrificed at 48 weeks post‐implantation, and liver and lung tissues were harvested for histological examination and metabolic analysis. The experimental timeline of the animal study is shown in Figure 6A.

### 2.9 Statistical analysis

Data were analyzed using SPSS 20.0 and GraphPad 8.0. Statistical significance was determined using a one‐way analysis of variance for multiple comparisons. Two‐sided Student's *t*‐test was used to compare the two groups. Overall survival was assessed using the Kaplan–Meier method and the log‐rank test. Clinicopathological characteristics of HCC patients were analyzed using *χ* ^2^ analysis. Data are expressed as mean ± standard deviation (SD). A *p‐*value <.05 was considered statistically significant.

Please refer to Supplementary Information for additional materials and methods used in this study.

## 3 RESULTS

### 3.1 GSTZ1 loss accelerates HCC metastasis both in vitro and in vivo

**FIGURE 1:**
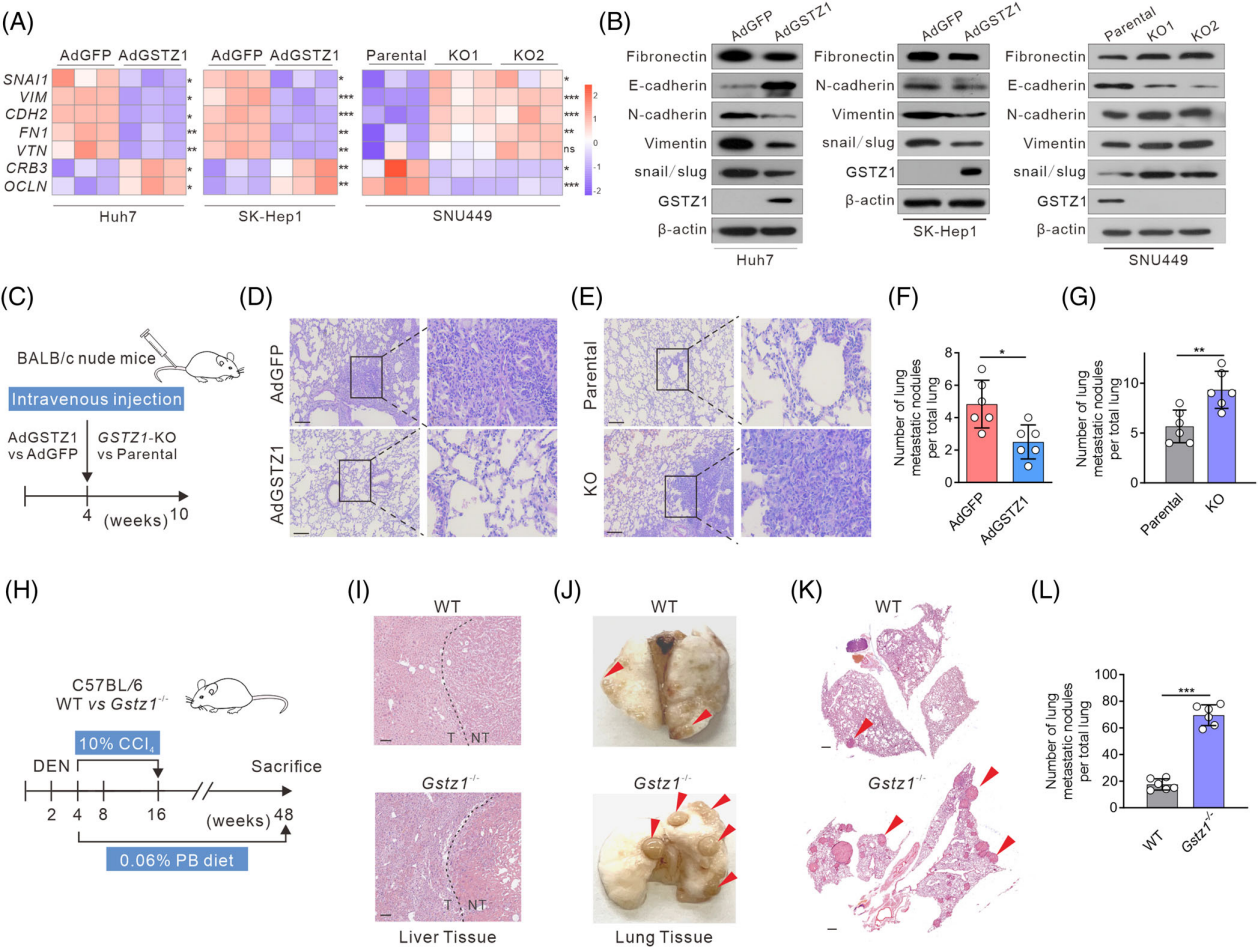
GSTZ1 loss accelerates hepatocellular carcinoma (HCC) metastasis both in vitro and in vivo. (A) Quantitative reverse transcription‐PCR (qRT‐PCR) analysis of epithelial‐to‐mesenchymal transition (EMT)‐related genes *SNAI1*, *VIM*, *CDH2*, *FN1*, *VTN*, *CRB3*, and *OCLN* in Huh7 or SK‐Hep1 cells infected with AdGSTZ1 or AdGFP, or GSTZ1‐KO SNU449 cells (*n* = 3). (B) Immunoblotting analysis of EMT‐related proteins. (C) Scheme for tail‐vein injection of GSTZ1‐OE Huh7 cells or GSTZ1‐KO SNU449 cells into randomized BALB/c nude mice. (D and E) Hematoxylin‐and‐eosin (H&E) staining of occult metastases in mouse lung tissue sections. Scale bar: 10 μm. (F and G) Number of lung metastases (*n* = 6 per group). (H and L) Scheme for diethylnitrosamine (DEN) and CCl_4_ treatment to induce HCC mouse model in C57BL/6J wild‐type (WT) and *Gstz1* ^−/−^ mice (H). PB, phenobarbital. H&E staining of mouse liver tissue (I). NT, non‐tumour; T, tumour. Representative images (J and K) and quantification (L) of lung metastasis. Scale bar: 50 μm. Data are mean ± SD. *p‐*Values were derived from an unpaired, two‐tailed Student's *t*‐test in (A); Mann–Whitney *U* test in (F, G and L) (*p < .05, **p < .01, ***p < .001).

To evaluate the role of GSTZ1 in HCC metastasis, we constructed a recombinant adenovirus encoding GSTZ1 (AdGSTZ1), and an adenovirus expressing green fluorescent protein (AdGFP) was used as a control. We generated CRISPR/Cas9‐mediated GSTZ1‐knockout (KO) SNU449 cell lines, KO1 and KO2(Figure S1A–C). Functional studies indicated that GSTZ1 overexpression (GSTZ1‐OE) significantly repressed the migration of both Huh7 and SK‐Hep1 cells, whereas GSTZ1 knockout promoted HCC cell migration compared with parental SNU449 cells, as shown in the transwell and wound healing assays (Figure S1D–I). EMT is a developmental process hijacked by cancer cells to acquire pro‐metastatic properties.^22^ Therefore, we examined the effects of GSTZ1 on EMT‐related molecules. GSTZ1 increased the expression of the epithelial junctional marker CDH1 (encoding E‐cadherin), OCLN and epithelial adhesion molecule CRB3, while decreasing the expression of the mesenchymal markers FN1 (which encodes fibronectin), VIM (which encodes vimentin) and SNAI1 (which encodes SNAIL). In contrast, GSTZ1 loss displayed a pronounced decrease in epithelial markers and increased mesenchymal markers (Figure 1A,B and Figure S1J,K). To determine whether GSTZ1‐KO cells would also show increased metastatic potential in vivo, we injected hepatoma cells into the tail veins of BALB/c nude mice (Figure 1C). In contrast to the AdGFP control, GSTZ1 overexpression significantly suppressed the metastatic potential of HCC cells, while GSTZ1 loss endowed hepatoma cells with the ability to colonize the lungs and form metastatic lesions (Figure 1D–G). We further determined the effect of *Gstz1* deletion on HCC metastasis using the well‐established DEN/CCl_4_ mouse model (Figure 1H). As shown in Figure 1I–L, *Gstz1* deletion (*Gstz1* ^−/−^) mice exhibited a higher metastatic ability and tumour burden than wild‐type (WT) mice, based on increased lung nodules (Figure 1J), larger and more numerous microscopic pulmonary lesions (Figure 1K,L), higher liver‐to‐body weight (LW/BW) ratios and higher serum aspartate aminotransferase (AST) activity (Figure S1L,M). Taken together, these studies indicate that GSTZ1 loss promotes HCC metastasis.

### 3.2 GSTZ1 deficiency enhances glucuronic pathway activity

**FIGURE 2:**
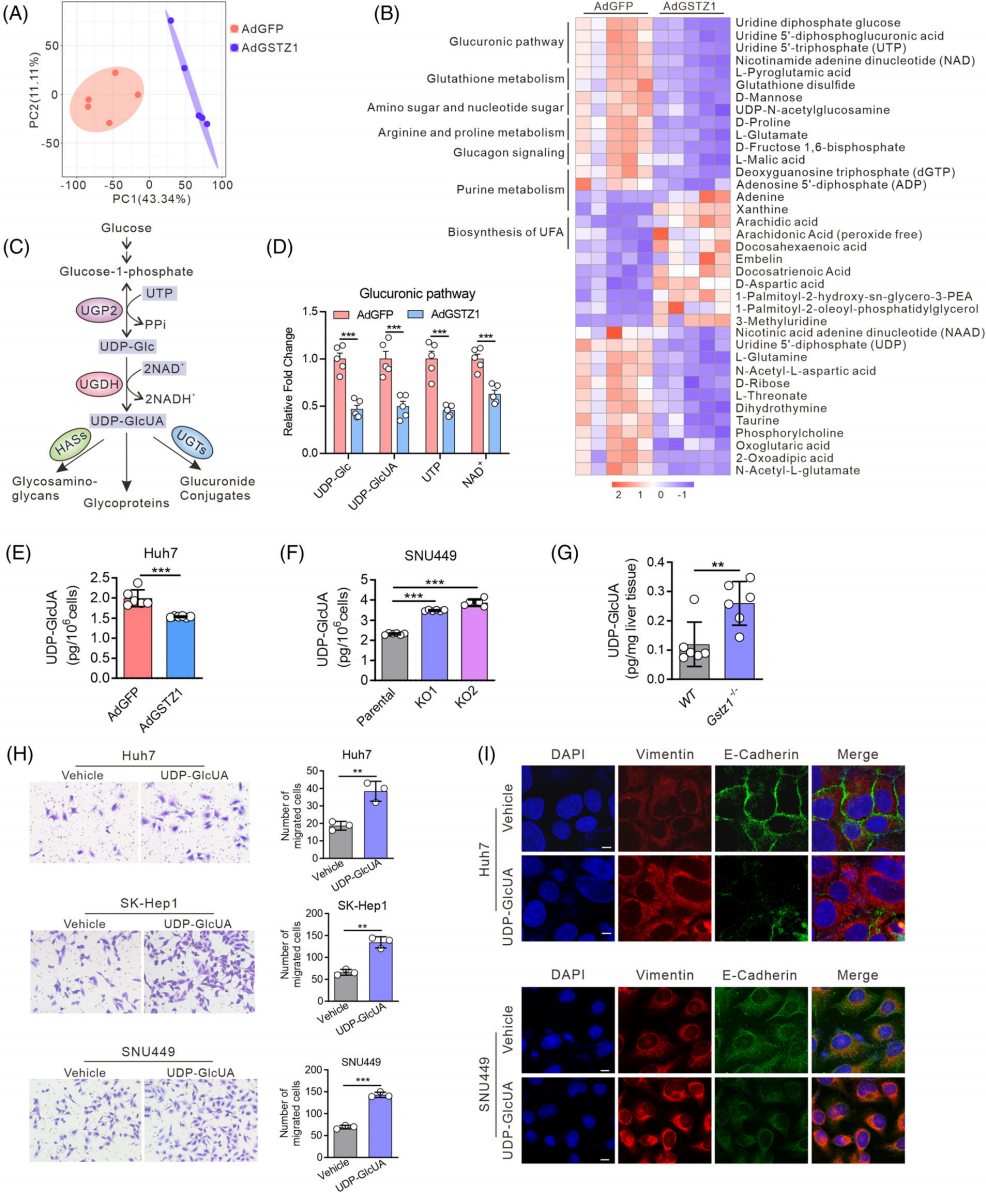
GSTZ1 deficiency enhances glucuronic pathway activity. (A) Principal component analysis of metabolite signatures in Huh7 cells infected with AdGSTZ1 or AdGFP using a metabolomics assay. PC, primary component. (B) Heatmap of differentially expressed metabolites subjected to identical treatment conditions as in (A). (C) Overview of the glucuronic pathway. (D) Relative changes in intermediate metabolites of the glucuronic pathway. (E–G) Uridine 5′‐diphosphate glucuronic acid (UDP‐GlcUA) levels in GSTZ1‐OE Huh7 cells (E), GSTZ1‐KO SNU449 cells (F) and *Gstz1* ^−/−^ mice liver tissues (G) quantified by the liquid chromatography‐tandem mass spectrometry (LC‐MS/MS)‐targeted metabolomics assay. (H) Transwell migration assays and quantification of the migrated cells in hepatoma cells supplemented with or without UDP‐GlcUA (0.5 mM for Huh7, 1 mM for SK‐Hep1, and 0.5 mM for SNU‐449) for 24 h. The migrated cells were stained with crystal violet staining. *n* = 3 independent experiments. (I) Representative immunofluorescence staining of E‐cadherin and vimentin from three independent experiments. Scale bar, 100 μm. Data are mean ± SD. *p‐*Values were derived from an unpaired, two‐tailed Student's *t*‐test in (D–F and H), and Mann–Whitney *U* test in (G) (**p < .01, ***p < .001).

To investigate the metabolic effects of GSTZ1, we performed a non‐targeted metabolomics comparison of GSTZ1‐OE and control Huh7 cells using liquid chromatography‐MS (Figure 2A). Thirty‐seven metabolites were significantly altered (two‐sided *t*‐test, *p* < .05) (Figure 2B). The pronounced decline in glutathione and l‐pyroglutamic acid (glutathione metabolism) and elevated arachidonic and arachidic acids (unsaturated fatty acid biosynthesis) were expected, considering the roles of GSTs in glutathione‐dependent reactions.^23^ Notably, glucuronic pathway‐related metabolites, including uridine diphosphate‐glucose (UDP‐Glc), UDP‐GlcUA and cofactors uridine triphosphate (UTP) and nicotinamide adenine dinucleotide (NAD^+^), were markedly decreased in GSTZ1‐OE huh7 cells (Figure 2B–D). To gain a deeper insight into the glucuronic pathway, we measured UDP‐Glc and UDP‐GlcUA levels using targeted metabolomics. Both UDP‐Glc and UDP‐GlcUA levels were consistently lower in GSTZ1‐OE cells (Figure 2E, and Figure S1N), whereas higher levels of UDP‐GlcUA but not UDP‐Glc were consistently found in GSTZ1‐KO cells (Figure 2F, Figure S1O) and *Gszt1* deletion mice (Figure 2G, Figure S1P). To test whether any of these metabolites were responsible for inducing pro‐aggressive effects, we treated hepatoma cells with UDP‐Glc or UDP‐GlcUA. Only UDP‐GlcUA but not UDP‐Glc (data not shown) induced pro‐migration effects (Figure 2H, Figure S2A–C) and an EMT‐like phenotype with a decline in E‐cadherin and a concurrent increase in vimentin and fibronectin (Figure 2I, Figure S2D–I). These data suggest that GSTZ1 deficiency enhances glucuronic pathway activity and induces pro‐aggressive phenotypes in HCC.

### 3.3 UGDH‐mediated UDP‐GlcUA accumulation promotes hepatoma cell migration upon GSTZ1 loss

UDP‐GlcUA is the active form of glucuronic acid involved in the biosynthesis of extracellular matrix GAG, various UDP‐sugars and the enzymatic glucuronidation, thereby playing an important role in diverse cellular processes such as matrix organization, morphogenesis, wound healing, inflammation, signalling, and tumour progression.^24^, ^25^, ^26^ To investigate which metabolic enzymes in this pathway contribute to UDP‐GlcUA accumulation, we analyzed the expression of metabolic enzymes including UDP‐glucose pyrophosphorylase 2, UGDH, UDP‐glucuronosyltransferase (UGT1A1), hyaluronan synthases (HAS1, ‐2, ‐3), hyaluronidases (HYAL1, ‐2) and UDP‐glucuronate decarboxylase 1(UXS1). As shown in Figure S3A,B, UGDH, a key rate‐limiting enzyme in the glucuronate pathway, which converts UDP‐Glc into UDP‐GlcUA, was the most up‐regulated metabolic enzyme in the liver tissues of *Gstz1*‐deletion mice. We further verified that UGDH was considerably decreased in GSTZ1‐OE cell models (Figure S3C,D) but increased in cells upon GSTZ1 knockout (Figure S3E), while other enzymes in the glucuronic pathway were not consistently altered. Furthermore, UGDH blockade using genetic approaches (sg*UGDH*) or pharmacological inhibitors (4‐MU) inhibited tumour cell migration, whereas supplementing UDP‐GlcUA fully restored the migratory capacity of UGDH‐depleted hepatoma cells (Figure S3F,I and Figure S3J–M), suggesting that accumulating the key intermediate metabolite UDP‐GlcUA, due to UGDH upregulation, may be critical for the pro‐metastatic phenotype mediated by GSTZ1 deficiency.

UGDH has been reported as a downstream target gene of NRF2 pathway.^27^, ^28^ Our previous experiments have demonstrated that GSTZ1 deficiency activates the NRF2 pathway^18^, ^19^ (Figure S4A). In this study, we found that the abilities of GSTZ1 deficiency‐mediated UGDH up‐regulation, UDP‐GlcUA accumulation, and tumour migration promotion were NRF2 dependent (Figure S4B–M).

### 3.4 GSTZ1 loss activates TGFβ/Smad signalling

**FIGURE 3:**
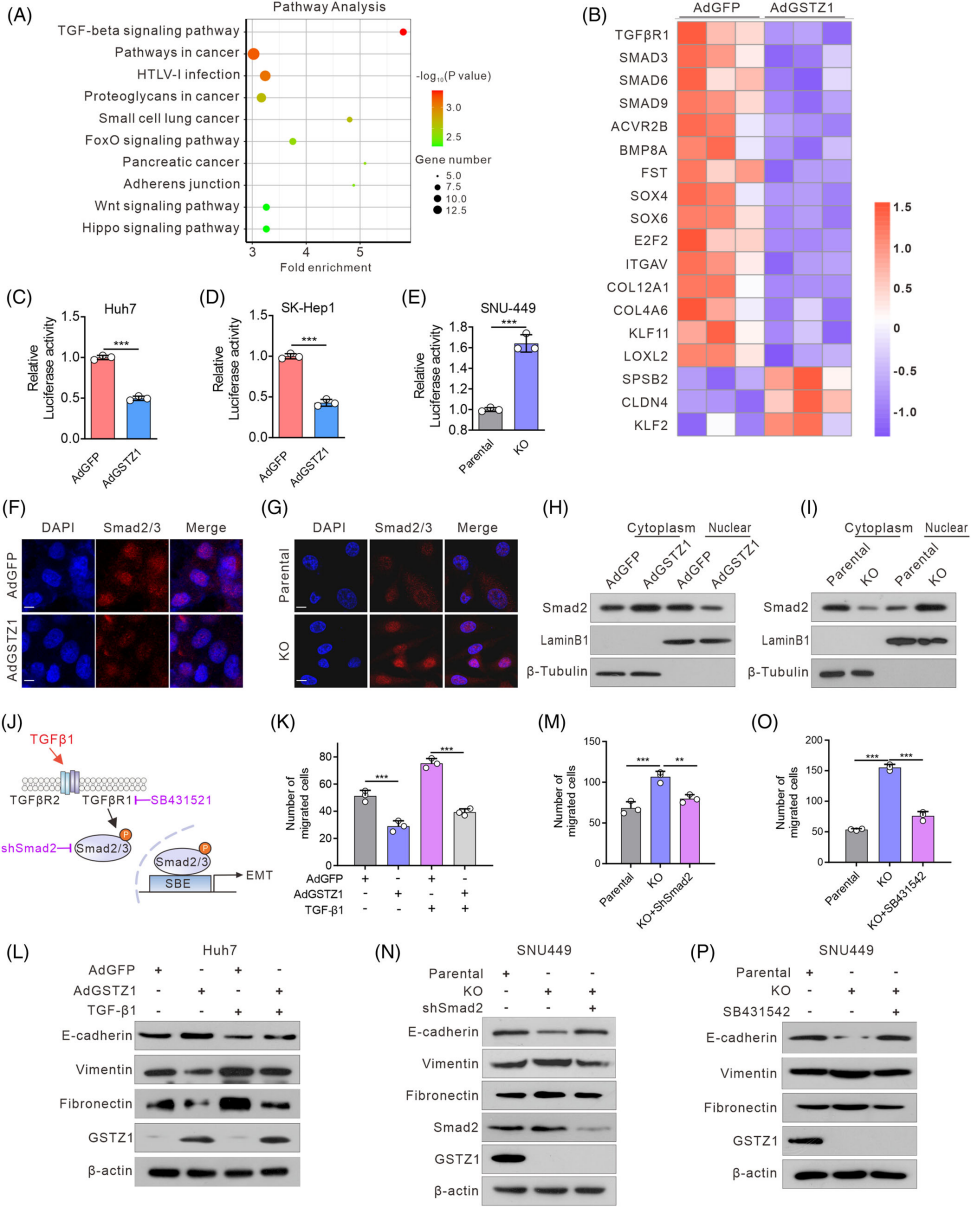
GSTZ1 loss activates transforming growth factor‐β/Smad signalling. (A) RNA sequencing analysis of top 10 significantly down‐regulated Kyoto Encyclopedia of Genes and Genomes (KEGG) pathways between AdGSTZ1‐ and AdGFP‐infected Huh7 cells (*n* = 3, each). (B) Heatmap of TGFβ/Smad pathway‐related differentially expressed genes (DEGs) identified based on the following criteria: false discovery rate < 0.05 and fold change > 1.5 or < 0.666. (C–E) TGFβ/Smad‐mediated gene transcriptional activities in GSTZ1‐OE (C and D) or ‐KO (E) Hepatocellular carcinoma (HCC) cells as assessed by smad binding element (SBE)‐luciferase assays (*n* = 3). (F and G) Intracellular localization of Smad2/3 (red) in GSTZ1‐OE Huh7 (F) or GSTZ1‐KO SNU‐449 cells (G). (H and I) Western blot analysis of cytoplasmic and nuclear Smad2/3 protein expression in GSTZ1‐OE Huh7 (H) or GSTZ1‐KO SNU‐449 (I) cells. β‐tubulin and laminB1 served as quality control for cytoplasmic and nuclear fractions, respectively. (J) Model of activation of the TGFβ/Smad pathway. (K and P) Quantification of the migrated cells and immunoblots of the epithelial‐to‐mesenchymal transition (EMT)‐related proteins in GSTZ1‐OE Huh7 cells supplemented with or without TGFβ1 (10 ng/ml, 24 h) (K and L), and GSTZ1‐KO SNU‐449 cells transfected with shSmad2 (M and N) or supplemented with SB431542 (10 μM, 36 h) (O and P). Data are mean ± SD. *p*‐Values were derived from an unpaired, two‐tailed Student's *t*‐test in (C–E); one‐way Analysis of Variance (ANOVA) followed by the Tukey test in (K, M, and O) (**p < .01, ***p < .001).

To investigate how GSTZ1 impairs the observed tumour metastasis, we performed a global transcriptomic analysis of GSTZ1‐OE Huh7 cells. A total of 513 differentially expressed genes were identified following GSTZ1 overexpression, suggesting that GSTZ1 induced marked transcriptional reprogramming. Furthermore, Kyoto Encyclopedia of Genes and Genomes analysis showed that GSTZ1 negatively regulates signaling pathways associated with tumour progression, such as TGFβ, FoxO, Wnt and Hippo signaling (Figure 3A). Among these genetic programs, TGFβ signalling is the most significantly down‐regulated, and several mRNAs of the TGFβ signaling components, such as TGFβR1, SMAD3, ‐6, ‐9 and SOX4, were down‐regulated (Figure 3A,B). Notably, TGFβ signaling is involved in many cellular processes, such as growth inhibition, extracellular matrix regulation, cell migration, invasion and the EMT process.^29^ We further confirmed the decrease in the mRNA levels of TGFβ signaling components (Figure S5A), which correlated with an inhibition of TGFβ signalling and decreased nuclear translocation of Smad2/3 in GSTZ1‐OE cells. In contrast, GSTZ1 knockout showed a concomitant activation of TGFβ signalling and increased nuclear translocation of Smad2/3 (Figure 3C–I, Figure S5B,C). To test whether activation of TGFβ signalling is critical for the pro‐aggressive effects mediated by GSTZ1‐deficiency (Figure 3J), we supplemented GSTZ1‐OE cells with recombinant human TGFβ1. In this case, the impaired migration capacity and EMT‐like phenotype were diminished or abolished. Conversely, either inhibition of the TGFβ receptor with SB431542 or Smad2 knockdown was sufficient to block pro‐aggressive properties and the EMT‐like phenotype in GSTZ1‐KO cells (Figure 3K–P, and Figure S5D–L). These data suggest that TGFβ signalling is indispensable for pro‐migration properties and the EMT‐like phenotype induced by GSTZ1 loss.

### 3.5 UDP‐GlcUA stabilizes *TGFβR1* mRNA by enhancing its binding to PTBP3 and activating TGFβ/Smad signalling

**FIGURE 4:**
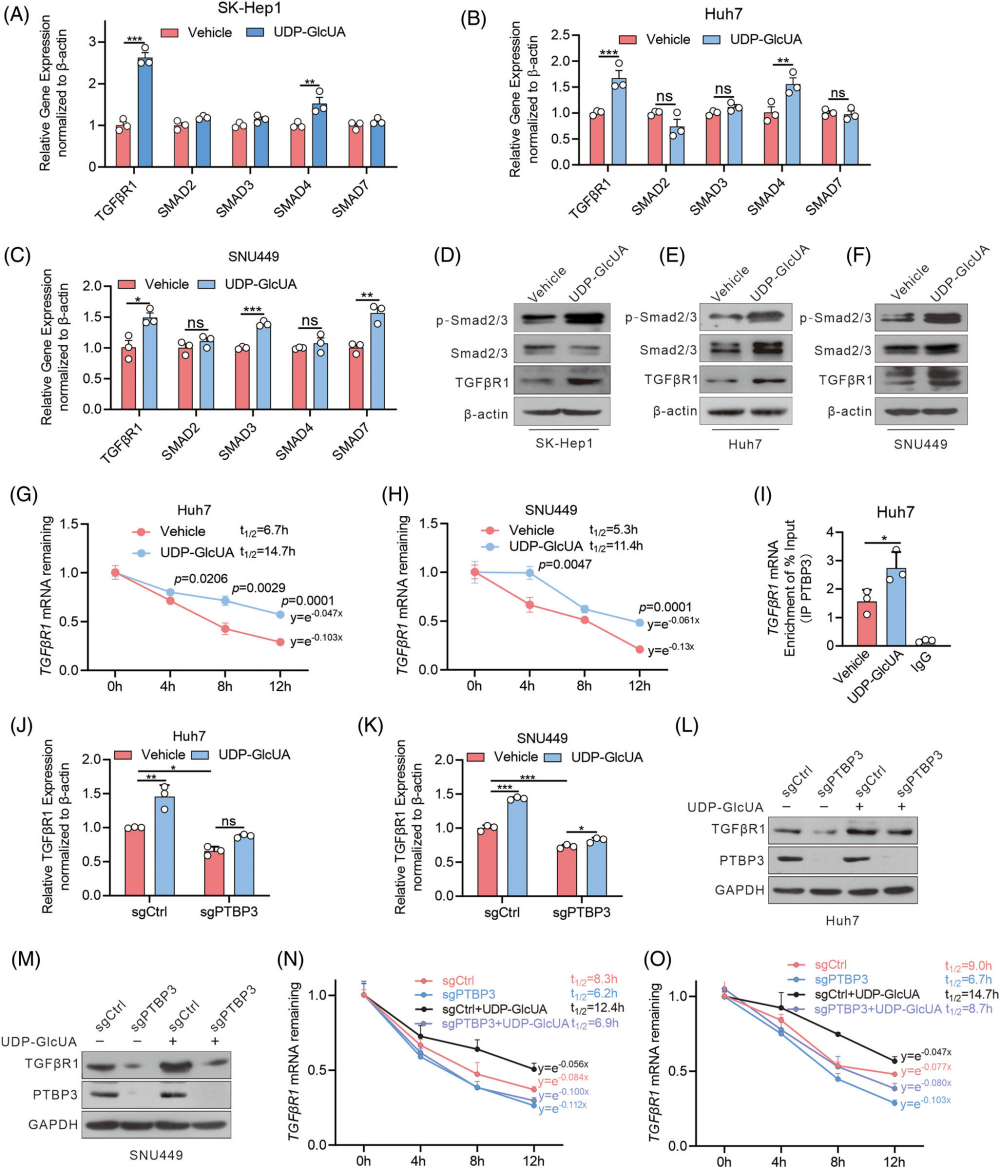
UDP‐GlcUA stabilizes *TGFβR1* mRNA and activates TGFβ/Smad signalling. (A–C) qRT‐PCR analysis of TGFβ/Smad pathway‐related genes (*n* = 3). (D–F) The expression of TGF‐β/Smad pathway‐related proteins by immunoblot. (G and H) *TGFβR1* mRNA half‐life in Huh7 and SNU449 cells supplemented with or without UDP‐GlcUA for 30 min before treated with DRB (50 μM) for indicated times (*n* = 3 independent experiments). DRB, 5,6‐dichloro‐1‐beta‐ribo‐furanosyl benzimidazole. (I) RNA Immunoprecipitation (RIP)‐qPCR showing the binding of PTBP3 to the *TGFβR1* in Huh7 cells supplemented with or without UDP‐GlcUA. IgG served as a negative control. (J–M) mRNA (J and K) and protein (L and M) expression levels of TGFβR1 in PTBP3‐depleted Huh7 and SNU449 cells after 0.5 h of UDP‐GlcUA treatment. (N and O) mRNA half‐life of TGFβR1 in PTBP3‐depletion Huh7 (N) and SNU449 (O) cells after 0.5 h of UDP‐GlcUA treatment. Data are mean ± SD. *p*‐Values were derived from an unpaired, two‐tailed Student's *t*‐test in (A–C), (G–I), (N–O) and one‐way ANOVA  followed by the Tukey test in (J and K) (*p < .01, **p < .01, ***p < .001).

There is growing evidence that metabolites exert signalling functions that contribute to tumourigenesis.^30^ Thus, we wondered whether GSTZ1 loss‐induced UDP‐GlcUA accumulation might modulate the TGFβ/Smad signalling pathway. To test this hypothesis, we treated Huh7, SK‐Hep1 and SNU449 cells with UDP‐GlcUA. Strikingly, UDP‐GlcUA elicited the rapid activation of the TGFβ/Smad pathway, including an increase in both the mRNA (Figure 4A–C) and protein (Figure 4D–F) levels of TGFβ/Smad signalling components, with TGFβR1 being one of the most highly up‐regulated genes. TGFβR1 activation is critical for the phosphorylation of R‐Smads, a key upstream event in canonical TGFβ signalling activation. TGFβR1 activation can be tightly regulated at multiple levels, including miRNA transcription,^31^ mRNA decay^32^ and protein degradation.^33^ Despite comparable TGFβR1 promoter activity (Figure S5M), UDP‐GlcUA supplementation exhibited enhanced *TGFβR1* mRNA stability (Figure 4G,H), suggesting that UDP‐GlcUA might post‐transcriptionally modulate TGFβR1.

RNA‐binding proteins (RBPs) are key components in RNA metabolism, and most RBPs recognize the specific sequence motifs frequently located within the 3′‐UTRs of their targets to regulate mRNA stability and translation. Thus, we screened for candidate RBPs potentially binding to the 3′‐UTR of *TGFβR1* mRNA according to the RBP suite database containing all human RBPs^34^ (Table S4). In total, 32 RBPs were identified, of which five were associated with HCC metastasis and progression: SFPQ, SAM68, ZRANB2, PTBP3 and SRSF1 (Figure S5N). RNA immunoprecipitation assays confirmed the association of polypyrimidine tract binding protein 3 (PTBP3), a nuclear‐enriched RBP also known as ROD1,^35^ with *TGFβR1* mRNA, while UDP‐GlcUA treatment further enhanced this association (Figure 4I), suggesting a potential regulatory link between UDP‐GlcUA and PTBP3‐targeting TGFβR1. Next, we determined whether PTBP3 regulates *TGFβR1* mRNA stability upon UDP‐GlcUA treatment. qRT‐PCR and immunoblot analysis confirmed that PTBP3 deletion disrupted TGFβR1 expression and stability. Most importantly, PTBP3 depletion abrogated UDP‐GlcUA‐increased TGFβR1 expression and its mRNA stability in hepatoma cells (Figure 4J–O), indicating that PTBP3 is essential for UDP‐GlcUA‐induced TGFβR1 expression.

**FIGURE 5:**
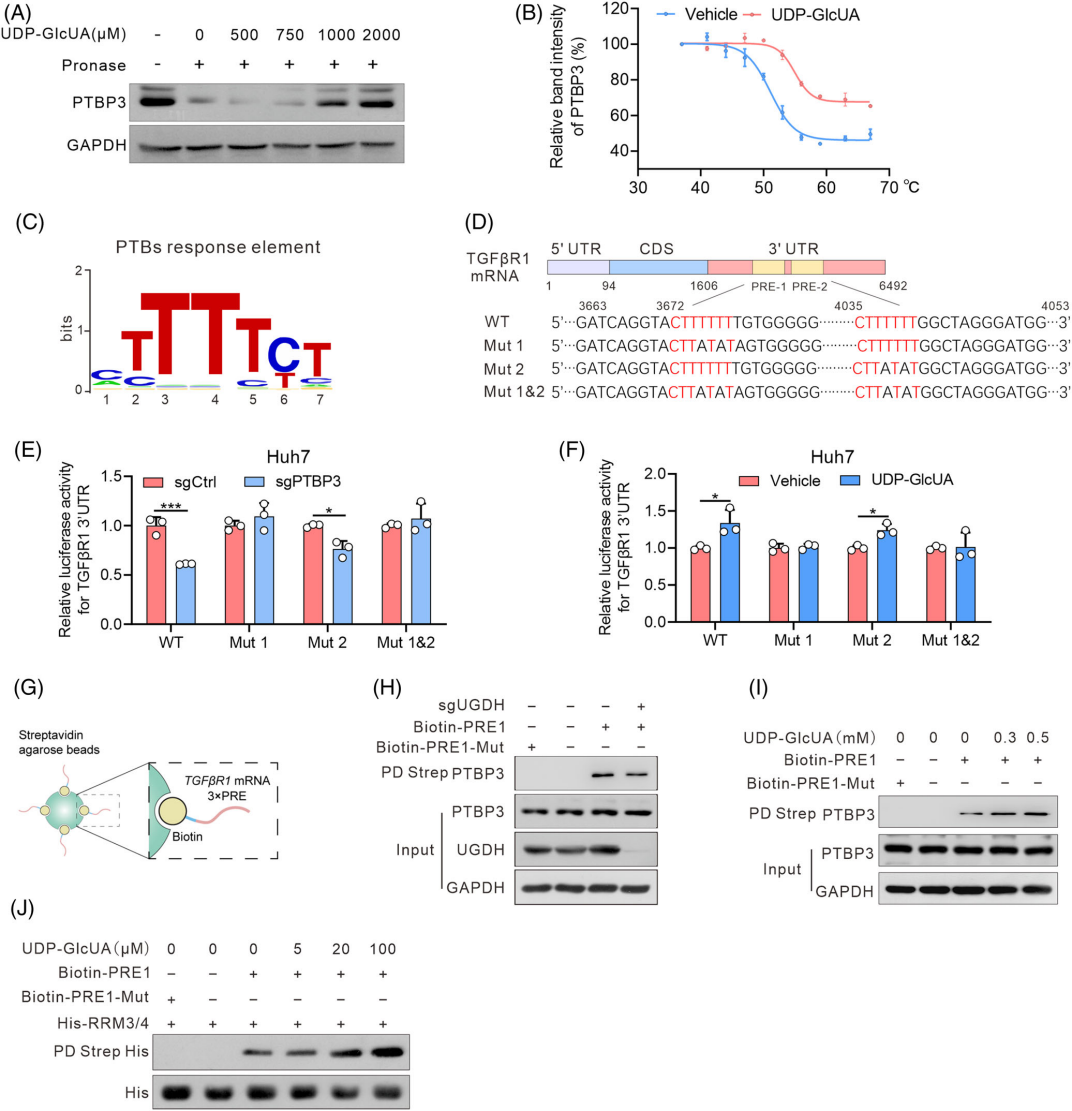
UDP‐GlcUA enhances *TGFβR1* mRNA binding of PTBP3. (A) DARTS assays with pronase digestion in Huh7 cells upon UDP‐GlcUA treatment. (B) Cellular thermal shift assay curves for PTBP3 in cell lysates supplemented with or without UDP‐GlcUA. Proteins denatured at the indicated temperature were probed with anti‐PTBP3, with GAPDH as a loading control. The bands were quantified using the Image‐Pro Plus analyzer software and normalized to the protein level of PTBP3 detected at 37°C (*n* = 3). (C) Conserved consensus PTBP3 response element (PRE). (D) Schematic drawing of two predicted consensus PTB response elements (PREs) in *TGFβR1* mRNA 3′‐UTR. WT for wild‐type, Mut 1 for PRE1 mutation, Mut 2 for PRE2 mutation, Mut 1 and Mut 2 for combo mutation. (E) Relative luciferase activities in Huh7 cells with or without PTBP3 depletion (*n* = 3 independent experiments). (F) Relative luciferase activities in Huh7 cells transfected with 3′‐UTR segments containing wild type (WT) or PRE‐1/2 mutants upon UDP‐GlcUA supplementation. (G–I) Streptavidin agarose affinity pull‐down assay using biotin‐PRE1 as a probe (G) in Huh7 cells with UDP‐glucose 6‐dehydrogenase (UGDH) depletion (H) or supplemented with UDP‐GlcUA (I). PRE1‐Mut served as a negative control. (J) Streptavidin agarose affinity pull‐down assay using biotin‐PREs (1 μg) as a probe by mixing purified recombinant His‐RRM3/4 (5 μg) and increasing doses of UDP‐GlcUA. Data are mean ± SD. *p*‐Values were derived from an unpaired, two‐tailed Student's *t*‐test in (B and E–F) (*p < .01, ***p < .001).

To determine if PTBP3 is a direct target of UDP‐GlcUA, we conducted DARTS and CETSA assays in hepatoma cells. Both assays demonstrated that PTBP3 protein stability is enhanced upon UDP‐GlcUA treatment, suggesting a potential direct binding between UDP‐GlcUA and PTBP3 (Figure 5A,B). PTBs recognize the cytosine uridylate‐rich sequences (PTB response elements, PREs) of mRNAs through four RNA recognition motifs (RRMs),^36^ with RRM3/4 being more functionally important because of an extended binding surface for RNA interaction provided by the ordered RRM3‐4 di‐domain packing.^37^ Sequence analysis revealed two putative PREs at positions 3672–3679 and 4035–4041 in TGFβR1 3′‐UTR, designated as PRE‐1 and PRE‐2(Figure 5C,D). To verify whether these two predicted PREs affect the post‐transcriptional regulation of *TGFβR1* mRNA by interacting with PTBP3, we constructed four TGFβR1 3′‐UTR fragments containing WT, or mutant PRE‐1 and/or 2 (Figure 5D). PTBP3 deletion greatly reduced the luciferase activity of the TGFβR1 3′‐UTR. However, the PRE‐1 mutation, but not the PRE‐2 mutation, substantially reversed the impaired luciferase activity mediated by PTPB3 deletion (Figure 5E). In addition, UDP‐GlcUA induced an increase in the luciferase activity of TGFβR1 3′‐UTR‐WT and ‐PRE‐2 mutations, but not the PRE‐1 or the combined PRE‐1 and PRE‐2 mutations (Figure 5F), suggesting that the PRE‐1 of TGFβR1 3′‐UTR is a key PTBP3 target site favored by UDP‐GlcUA.

Based on the above results, we performed a pull‐down assay on streptavidin beads to confirm the interaction sites between PTBP3 and TGFβR1 in response to UDP‐GlcUA with synthesized biotin‐labelled PREs (Figure 5G). Interestingly, PRE1 but not PRE1‐Mut interacted with PTBP3, and UGDH depletion impaired the binding of PTBP3 to PRE1 (Figure 5H), whereas UDP‐GlcUA increased the binding of PTBP3 to PRE1 in a dose‐dependent manner (Figure 5I). Similar results were observed in a pull‐down assay on streptavidin beads with biotin‐labelled PRE1 and purified recombinant His‐PTBP3‐RRM3/4 (Figure 5J).

Collectively, these data indicate that UDP‐GlcUA stabilizes *TGFβR1* mRNA by enhancing its binding of PTBP3, thereby activating TGFβ/Smad signalling.

### 3.6 Blockage of the glucuronic pathway or TGFβ signalling blunts HCC metastasis driven by *Gstz1* loss

**FIGURE 6:**
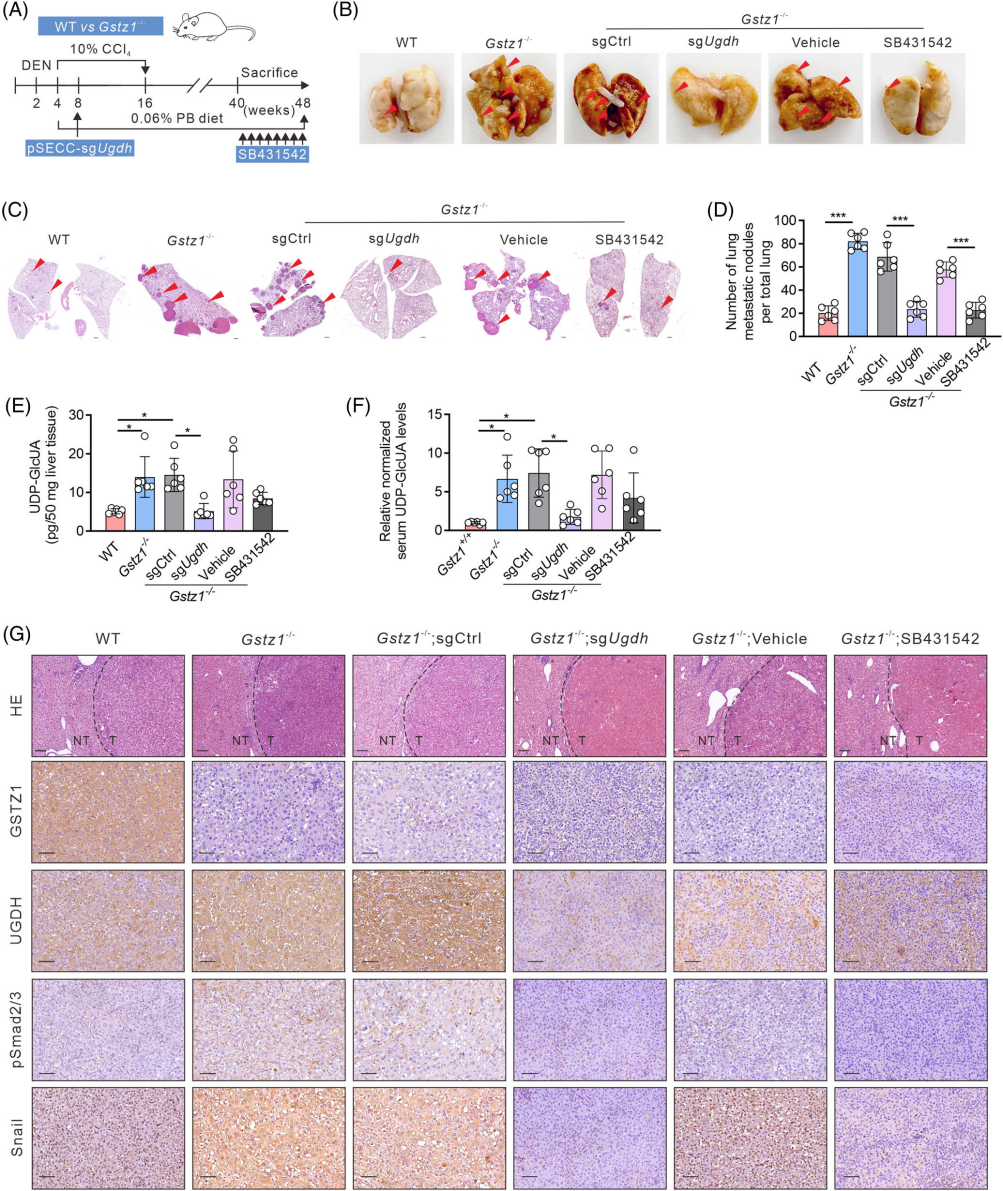
Blockage of the glucuronic pathway or TGFβ signalling blunts hepatocellular carcinoma (HCC) metastasis driven by *Gstz1* loss. (A) Schematic representation of diethylnitrosamine (DEN) and CCl_4_‐ induced mouse model of HCC. PB, phenobarbital. (B) Representative images of lung metastasis. (C) Hematoxylin‐and‐eosin (H&E) staining of occult metastases in lung tissue sections. Scale bar, 500 μm. (D) Number of lung metastases. Data represent mean ± SD of the relative number of nodules per mouse for six mice. (E) UDP‐GlcUA levels in mouse liver tissues. *n* = 6. (F) The relative content of UDP‐GlcUA normalized to the average UDP‐GlcUA level in serum samples obtained from *Gstz1* ^+/+^ mice . *n* = 6. (G) Hematoxylin‐and‐eosin (H&E) and Immunohistochemistry (IHC) staining for GSTZ1, UGDH, pSmad2/3 and Snail in WT and *Gstz1^−^* ^/‐^ mouse liver sections. NT, non‐tumour; T, tumour. Scale bar: 50 μm. Data are mean ± SD. *p*‐Values were derived from a one‐way ANOVA followed by the Tukey test in (D, E and F) (*p < .05, ***p < .001).

Given the contribution of the enhanced glucuronate pathway and TGFβ signalling to hepatoma cell migration upon GSTZ1 knockout, we determined whether blockage of the glucuronate pathway or TGFβ signalling dampens *Gstz1*‐deletion dependent tumour promotion. Exploiting the DEN/CCl_4_‐induced mouse model of HCC, we intravenously delivered pSECC‐sg*Ugdh* particles to delete *Ugdh* in *Gstz1* ^−/−^ mice (*Gstz1^−/−^*/sg*Ugdh*), with pSECC‐sgCtrl‐injected mice serving as controls (*Gstz1^−/−^*/sgCtrl) (Figure 6A). *Ugdh* deletion in *Gstz1* ^−/−^ mouse livers was validated by decreased protein levels. We also administered SB431542 to *Gstz1* ^−/−^ mice to inhibit TGFβ signalling at a later stage (*Gstz1^−/−^*/SB431542), with vehicle‐injected mice serving as controls (*Gstz1^−/−^*/Vehicle) (Figure 6A). As shown in Figure S6A, liver tumours were observed in mice from all groups at 48 weeks, with *Gstz1^−/−^*/sgCtrl livers exhibiting more and larger surface tumours, higher LW/BW ratios and serum AST activity (Figure S6B–D). In addition, *Gstz1^−/−^*/sg*Ugdh* or *Gstz1^−/−^*/SB431542 animals exhibited significantly fewer lung nodules (Figure 6B), smaller and fewer microscopic pulmonary lesions (Figure 6C,D), reduced liver tissue and serum UDP‐GlcUA levels (Figure 6E,F). Importantly, concomitant inhibition of TGFβ signalling and impaired EMT‐like phenotype were also observed in *Gstz1^−/−^*/sg*Ugdh* or *Gstz1* ^−/−^/SB431542 mice (Figure 6G and Figure S6E). Altogether, targeted blockage of the glucuronic pathway and TGFβ signalling blunted HCC metastasis in the *Gstz1*‐deletion mouse model.

### 3.7 GSTZ1 deficiency with UGDH up‐regulation promotes HCC metastasis

**FIGURE 7:**
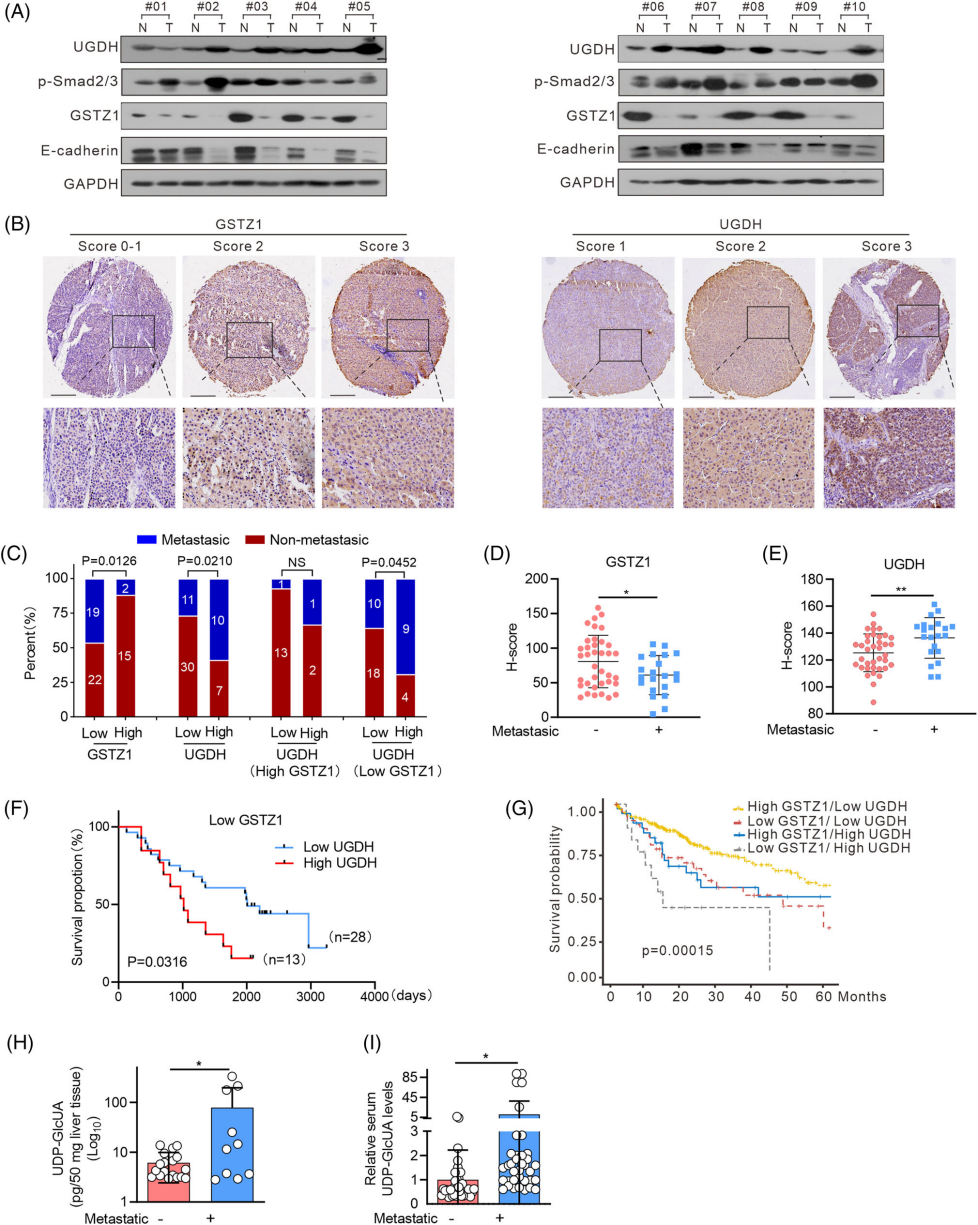
GSTZ1 deficiency with UGDH up‐regulation promotes hepatocellular carcinoma (HCC) metastasis. (A) Western blotting for UGDH, pSmad2/3, GSTZ1 and E‐cadherin in tumour tissues from HCC patients with metastatic recurrence and adjacent non‐tumoural tissues. (B) Representative Immunohistochemistry (IHC) staining of GSTZ1 and UGDH in HCC tissue microarray. Scores (ranging 0–3) were calculated by intensity and percentage of stained cells. Scale bar, 200 μm. (C) Percentage of metastatic or non‐metastatic recurrence of HCC patients (*n* = 58 samples), stratified by GSTZ1 and UGDH expression. Specifically, HCC patients were classified into low (scores of 5–92, 41 cases) versus high (scores of 93–159, 17 cases) GSTZ1 expression and low (scores of 89–138, 41 cases) versus high (scores of 139–162, 17 cases) UGDH expression subgroups. (D and E) Staining scores of GSTZ1 and UGDH in tumour tissues from HCC patients with metastatic recurrence (*n* = 21) and without metastatic recurrence (*n* = 37). (F) Kaplan–Meier survival analysis of overall survival rate for HCC patients in GSTZ1 low subgroup stratified by UGDH expression, using tissue microarray cohort. (G) Kaplan–Meier survival analysis of overall survival rate for patients with HCC from The Cancer Genome Atlas (TCGA)‐liver hepatocellular carcinoma dataset (*n* = 365), stratified by GSTZ1 and UGDH expression. (H) UDP‐GlcUA levels in tumour tissues from HCC patients with (*n* = 10) or without (*n* = 18) metastatic recurrence. (I) UDP‐GlcUA levels in serum samples from HCC patients with (*n* = 36) or without (*n* = 34) metastatic recurrence. Data are mean ± SD. *p‐*Values were derived from a chi‐square test in (C); an unpaired, two‐tailed Student's *t*‐test in (D and E); a two‐sided log‐rank test in (F–G), and Mann–Whitney *U* test in (H and I). (*p < .05, **p < .01, ***p < .001).

To define the clinical relevance of our findings, we determined the levels of UGDH, GSTZ1, pSmad2/3 and E‐cadherin in tumours from 10 HCC patients with metastatic recurrence. Lower GSTZ1 and E‐cadherin levels and higher UGDH and pSmad2/3 levels were observed in tumour samples than in adjacent non‐tumoural tissues (Figure 7A). This indicated that GSTZ1 deficiency concomitant with UGDH upregulation and TGFβ pathway activation might contribute to HCC metastasis. Next, we performed immunohistochemical analyses in a tissue microarray of 58 human HCC specimens, where patients were stratified by low versus high GSTZ1 and/or UGDH (Figure 7B). Notably, low GSTZ1, high UGDH and low GSTZ1 coupled with high UGDH were strongly associated with HCC metastasis (Figure 7C–E). Furthermore, patients with high UGDH expression (13 cases) had a significantly lower median length of survival (34 months) than those with low UGDH levels (28 cases, 67 months) in the subgroup of patients with low GSTZ1 (Figure 7F). Likewise, patients with low GSTZ1 together with high UGDH expression displayed the lowest median length of survival based on data from The Cancer Genome Atlas (TCGA) liver hepatocellular carcinoma dataset (Figure 7G). Importantly, elevated UDP‐GlcUA levels were observed in both tumour tissues and serum samples from patients with metastatic recurrence (Figure 7H,I). In summary, GSTZ1 deficiency and UGDH up‐regulation correlate with increased metastatic potential and decreased patient survival in HCC. Thus, increased circulating UDP‐GlcUA levels might be useful as a marker for poor prognosis in patients with HCC.

## 4 DISCUSSION

**FIGURE 8:**
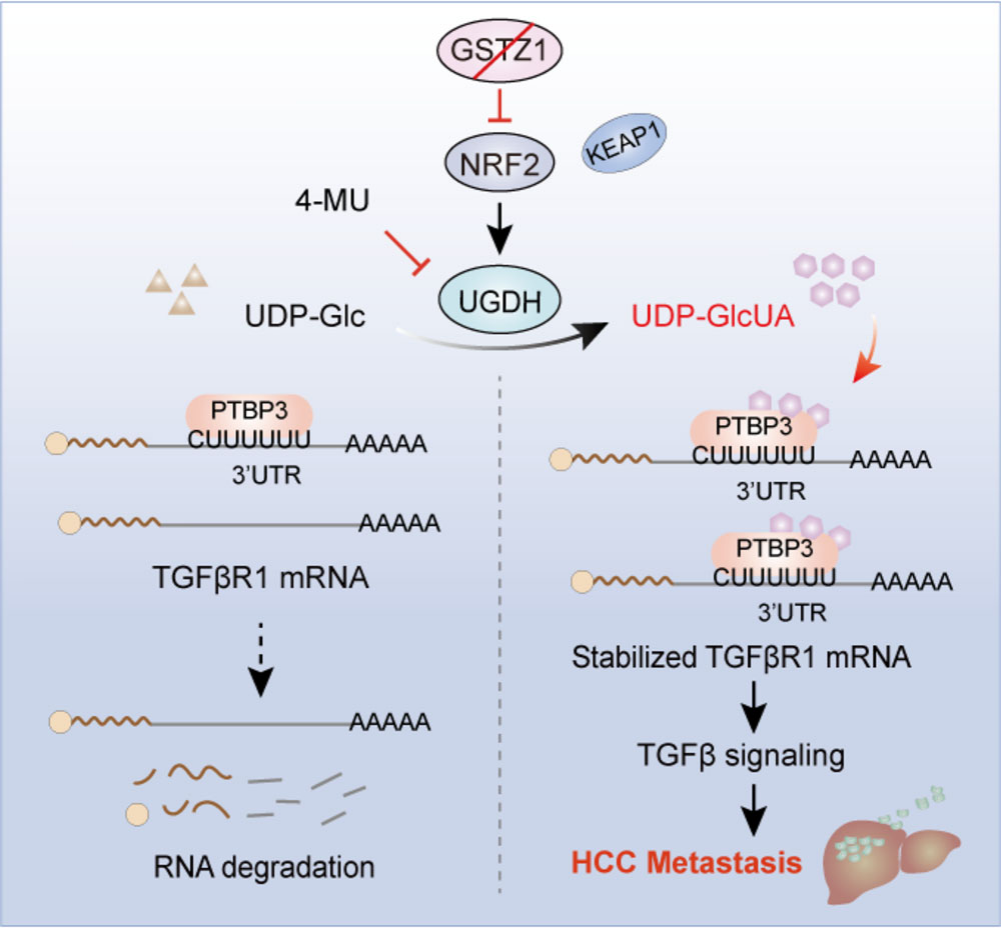
Proposed working model of this study. UDP‐glucose 6‐dehydrogenase (UGDH)‐mediated UDP‐GlcUA accumulation promotes hepatoma cell migration upon GSTZ1 loss. UDP‐GlcUA stabilized TGFβR1 mRNA by enhancing its binding to PTBP3, contributing to the activation of TGFβ/Smad signalling.

Our study provides evidence demonstrating that GSTZ1 loss facilitates HCC metastasis by coupling glucuronic acid metabolism to TGFβ/Smad signalling. This is initiated by reprogramming of glucuronic acid pathway upon GSTZ1 loss, thereby UDP‐GlcUA accumulation enhances *TGFβR1* mRNA stability, ultimately promoting HCC progression. Of note, we revealed that PTBP3 is a novel RBP critical for *TGFβR1* mRNA stability, thus exerting a pro‐metastatic role during TGFβ/Smad signalling‐induced EMT and HCC progression (Figure 8). These findings broaden our horizons regarding how metabolic pathways are involved in tumour progression and metastasis via signalling pathway modulation.

Normally produced by the healthy liver, UDP‐GlcUA is among the most powerful natural detoxifiers and is involved in the biosynthesis of extracellular matrix GAG. UGDH has been previously reported as the sole enzyme catalyzing UDP‐GlcUA production in humans.^38^ Dysregulated UGDH has been shown to promote epithelial cancer progression predominantly due to accumulation of UDP‐GlcUA and subsequent hyaluronan synthesis^39^, ^40^ and correlates with poor prognosis and chemoresistance in multiple cancers.^14^, ^41^, ^42^ In the present study, we found that GSTZ1 loss critically drives HCC metastasis by enhancing glucuronic pathway activity. Surprisingly, elevated UDP‐GlcUA levels mediated by GSTZ1 loss were not due to the differential expression of metabolic enzymes involved in glucuronidation reactions or proteoglycan production but were primarily attributed to UGDH up‐regulation, a rate‐limiting and essential step in the glucuronic pathway. This could indicate that the UGDH‐regulated availability of UDP‐sugar precursors contributes to the pro‐metastatic phenotype in HCC. Furthermore, UGDH deletion impaired lung cancer metastasis through the UDP‐Glc‐mediated destabilization of *SNAI1* mRNA,^14^ suggesting the roles of different metabolites that accumulated primarily due to aberrant UGDH function in facilitating the EMT process and tumour metastasis, which may be multiple and context‐dependent.

Interestingly, an increase in several members of the TGFβ/Smad pathway associated with UDP‐GlcUA accumulation was observed, suggesting a link between the activation of TGFβ signalling and the acquisition of pro‐metastatic properties underlying UDP‐GlcUA accumulation. Indeed, genetic or pharmacological targeting of UGDH in lung, ovarian and breast cancer models significantly impaired tumour metastasis.^13^, ^14^, ^43^ UGDH inhibitors, such as 4‐MU and quercetin, have also received increased attention in potential cancer therapeutic strategies.^44^, ^45^, ^46^, ^47^ Here, we show that *Ugdh* deletion significantly reduces HCC metastasis in *Gstz1*‐deletion mice. Moreover, these findings may be relevant for human HCC. UGDH up‐regulation correlates with increased metastatic potential and decreased survival of patients with HCC, especially in those with low GSTZ1 expression. In addition, elevated UDP‐GlcUA levels in both serum and tumour tissues were observed in patients with metastatic recurrence, which may represent a potential biomarker for the prognosis stratification of HCC patients. However, whether serum UDP‐GlcUA is directly related to UGDH expression in HCC tissue and has therapeutic and prognostic significance for HCC requires further investigation.

Aberrant induction of the TGFβ pathway is strongly linked to tumour metastasis.^48^ As one of the central molecular mediators of TGFβ signalling, TGFβ receptors are tightly regulated by post‐translational modifications, miRNAs, as well as by interaction with other proteins.^49^, ^50^ However, the RBPs‐mediated post‐transcriptional mechanisms that control TGFβ signalling are poorly understood. A recent report has demonstrated that Quaking 5 (QKI‐5) mediates post‐transcriptional degradation of *TGFβR1* mRNA, thereby inhibiting TGFβ/Smad signalling in lung adenocarcinoma cells.^32^ Here, we identified another RBP, PTBP3, as an activator of TGFβR1. Specifically, PTBP3 could directly interact with the *TGFβR1* 3′‐UTR and stabilize *TGFβR1* mRNA, consequently leading to the activation of TGFβ signalling. Indeed, various cellular stresses such as hypoxia and hyperglycemia are known to stimulate the binding of PTBs to the 3′‐UTR of their target mRNAs,^51^, ^52^ achieved by regulating its intracellular localization, conformation changes,^53^ or its associations with other factors such as cold shock domain Y‐box proteins.^54^ Interestingly, we found that UDP‐GlcUA facilitated the binding of PTBP3 to *TGFβR1* mRNA, indicating a novel level of post‐transcriptional regulation by coupling metabolic signalling and RBPs. In the future, it will be interesting to test whether glucuronic acid metabolism affects other signalling pathways, and how a metabolite such as UDP‐GlcUA modulates cellular phenotypes and activities by regulating mRNA stability.

## CONFLICT OF INTEREST

The authors have declared that no conflict of interest exists.

## Supporting information

Supplement Material
