# Corrigendum to “Dysregulated glucuronic acid metabolism exacerbates hepatocellular carcinoma progression and metastasis through the TGFβ signalling pathway”

**DOI:** 10.1002/ctm2.1231

**Published:** 2023-03-20

**Authors:** Qingzhu Gao, Bin Cheng, Chang Chen, Chong Lei, Xue Lin, Dan Nie, Jingjing Li, Luyi Huang, Xiaosong Li, Kai Wang, Ailong Huang, Ni Tang

Gao Q, Cheng B, Chen C, et al. Dysregulated glucuronic acid metabolismexacerbates hepatocellular carcinoma progressionand metastasis through the tumour growth factor beta signalling pathway. *Clin Transl Med*. 2022;12:e995. https://doi.org/10.1002/ctm2.995

In this article, Figure 6G were inadvertently assembled by errors. We have now updated the staining image for Snail in the “*Gstz1^−/−^*; sg*Ugdh*” group in Figure 6G. The corrected Figure 6G is as follows.

**FIGURE 6:**
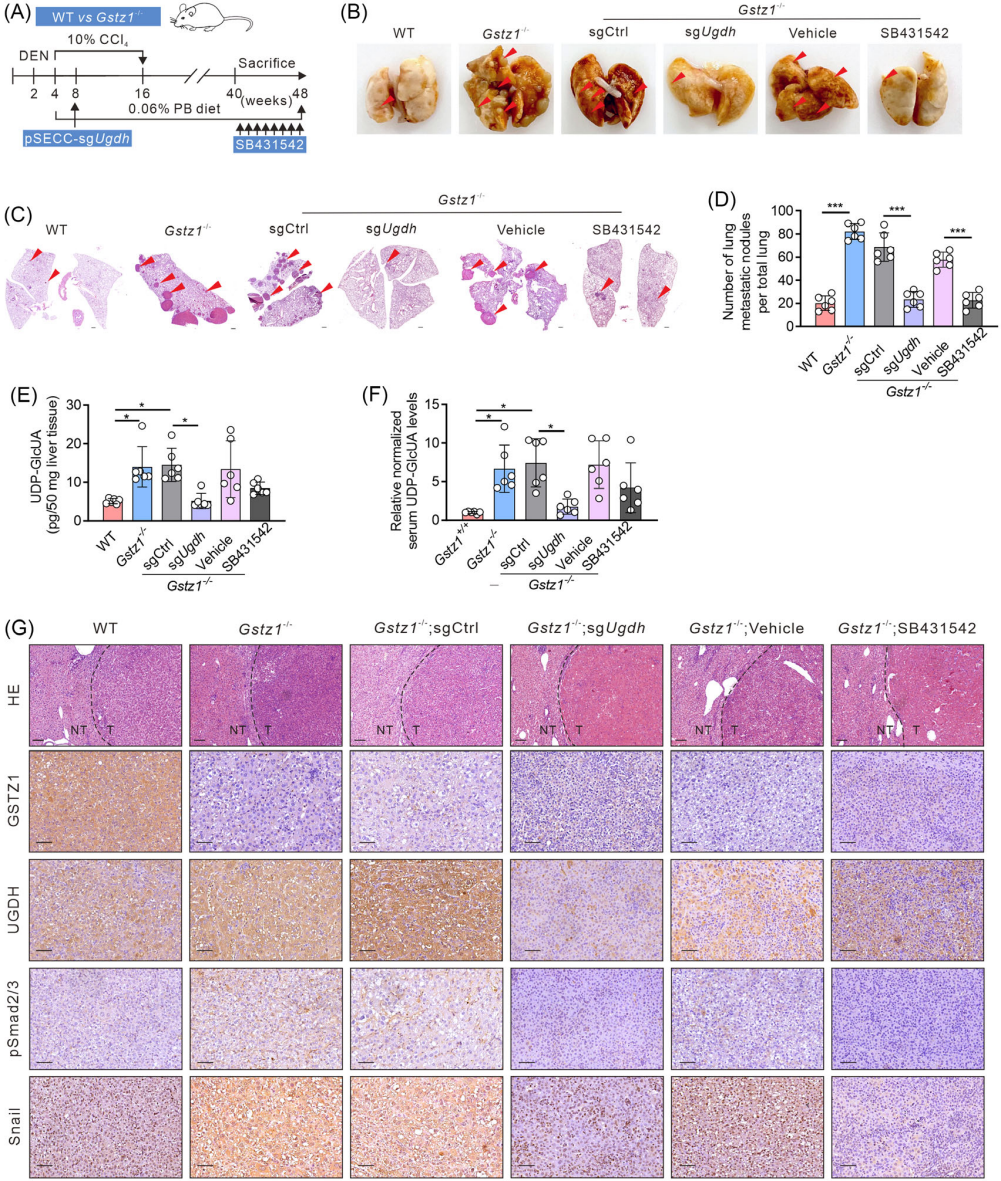
Blockage of the glucuronic pathway or tumour growth factor beta (TGFβ) signalling blunts hepatocellular carcinoma (HCC) metastasis driven by *Gstz1* loss. (A) Schematic representation of diethylnitrosamine (DEN) and CCl_4_‐ induced mouse model of HCC. PB, phenobarbital. (B) Representative images of lung metastasis. (C) Hematoxylin‐and‐eosin (H&E) staining of occult metastases in lung tissue sections. Scale bar, 500 µm. (D) Number of lung metastases. Data represent mean ± SD of the relative number of nodules per mouse for six mice. (E) UDP‐GlcUA levels in mouse liver tissues. *n* = 6. (F) The relative content of UDP‐GlcUA normalized to the average UDP‐GlcUA level in serum samples obtained from *Gstz1* ^+/+^ mice . *n* = 6. (G) Hematoxylin‐and‐eosin (H&E) and Immunohistochemistry (IHC) staining for GSTZ1, UGDH, pSmad2/3 and Snail in WT and *Gstz1^−^* ^/−^ mouse liver sections. NT, non‐tumour; T, tumour. Scale bar: 50 µm. Data are mean ± SD. *p*‐Values were derived from a one‐way analysis of variance (ANOVA) followed by the Tukey test (D–F) (*p < .05, ***p < .001).

The author apologizes for this error move this after the artwork and figure caption.

