# Supplementary material for: Dysregulated glucuronic acid metabolism exacerbates hepatocellular carcinoma progression and metastasis through the TGFβ signalling pathway
Source: Clin Transl Med. 2022 Aug 17;12(8):e995. doi: 10.1002/ctm2.995 (PMC9386326; doi:10.1002/ctm2.995)
Supplement: Supplementary file 1 — Supplement Material [file CTM2-12-e995-s001.docx]

**Dysregulated glucuronic acid metabolism exacerbates hepatocellular carcinoma progression and metastasis through the TGFβ signaling pathway**

Qingzhu Gao^1,#^, Bin Cheng^1,#^, Chang Chen^2,#^, Chong Lei^1,#^, Xue Lin^1^, Dan Nie^3^, Jingjing Li^1^, Luyi Huang^1^, Xiaosong Li^4^, Kai Wang*^1^, Ailong Huang*^1^, Ni Tang*^1^

**Methods**

**Supplementary Fig. 1. GSTZ1 inhibits hepatocellular carcinoma (HCC) metastasis and glucuronate pathway activity.**

**Supplementary Fig. 2. UDP-GlcUA promotes hepatoma cell migration and epithelial-mesenchymal transitions.**

**Supplementary Fig. 3. UGDH-mediated UDP-GlcUA accumulation promotes hepatoma cell migration upon GSTZ1 loss**

**Supplementary Fig. 4. GSTZ1 inhibits glucuronate pathway activity via the NRF2/UGDH axis.**

**Supplementary Fig. 5. GSTZ1 inhibits transforming growth factor-β/Smad signaling.**

**Supplementary Fig. 6. Blockage of the glucuronic pathway or TGFβ signaling blunts hepatocellular carcinoma (HCC) metastasis driven by Gstz1 loss.**

**Table S1. Primer sequences used in this study.**

**Table S2. Antibodies used in this study.**

**Table S3. Chemicals, Critical Commercial Assays and Experimental**

**Models.**

**Table S4. 154 RBPs through eCLIP-seq from ENCODE corresponding to**

**human genome hg19 version.**

**Methods**

**Drug treatments**

For the in vitro assays, 250 mM 4-Methylumbelliferone (4-MU, MedChemExpress, HY-N0187), 1 mM brusatol (MB7292; Dalian Meilunbio Co. Ltd., Dalian, China), and 10 mM SB431542, all dissolved in 100% dimethyl sulfoxide (DMSO), were used as stock solutions. Cells were treated at a final concentration of 1 to 2% DMSO with 500 µM 4-MU, 60 nM brusatol, 10 µM SB431542, or untreated vehicle control.

**Adenoviruses and reporter plasmids**

The recombinant adenoviral plasmids pAd-GSTZ1 were constructed as previously described [^1^](#_ENREF_1)^,^ [^2^](#_ENREF_2), AdGFP ( an analogous adenovirus expressing only GFP) was used as control. PCR-amplified GSTZ1 (NM_145870.2) or, KEAP1 (NM_012289.3) was cloned into pSEB-3Flag vector (a gift from Prof. T-C He, University of Chicago, USA). The 3′ UTR of TGFβR1 (NM_004612.4) containing PTBP3 binding sites from 3520-4221 bp were amplified from human genome DNA, digested with *Nhe*I, and inserted into the *Xba*I site of pGL3-Basic (Promega); TGFβR1-3′ UTR mutations were generated by site-directed mutagenesis. The wild-type TGFβR1-3′ UTR plasmids were used as a template. For expression of His-tagged recombinant human PTBP3 proteins, the PTBP3 ORF sequences were cloned into the N-terminal 6× His-tagged pET-28a vector. The primer sequences used for PCR amplification are provided in Table S1.

**CRISPR/Cas9-meditated gene knockout**

LentiCRISPR v2 *GSTZ1* sgRNA, *UGDH* sgRNA or *PTBP3* sgRNA was constructed with the oligonucleotide targeting the coding region of the corresponding gene (oligonucleotide sequences are provided in Table S1). GSTZ1 knockout (KO) SNU-449 cell lines were constructed using the CRISPR/Cas9 system as described previously [^3^](#_ENREF_3).

The pSECC vector was purchased from Addgene (# 60820). pSECC *Ugdh* sgRNA was generated with the oligonucleotide 5′ -GAAGTAGTCGAATCCTGT CG-3′, which targets the coding region of the mouse *Ugdh* transcript. For sgRNA cloning, the pSECC vector was digested with *Bsm*BI and ligated with *Bsm*BI-compatible annealed oligos. Lentivirus was generated by co-transfecting HEK293T cells with pSECC-sgUgdh, envelop plasmid pMD2.G, and packaging plasmid psPAX2 using Lipofectamine 3000 (Invitrogen, Carlsbad, CA, USA). Supernatant was collected 48 hrs post-transfection, concentrated by ultracentrifugation at 120,000 g for 6 hrs and resuspended in an appropriate volume of phosphate-buffered saline (PBS) [^4^](#_ENREF_4).

**Luciferase reporter assay**

Huh7 or GSTZ1-KO SNU-449 cells were transfected with 3 μg of pGL3-SBE-Luc (provided by Dr. T-C He, University of Chicago, USA) and 100 ng/well pRL-TK (an internal control) using Lipofectamine 3000. After 24 h, Huh7 cells were infected with AdGSTZ1 or vector control (AdGFP). Cells were harvested at 36 h post-infection, and analyzed for luciferase activity by dual-luciferase assay (Promega, Madison, WI, USA).

To determine the effect of PTBP3 on the luciferase activity of TGFβR1 3′-UTR, the WT or mutated TGFβR1 construct 3′-UTRs, pRL-TK were co-transfected into Huh7 cells with PTBP3 deletion. Luciferase activity was measured as previously described. All experiments were performed in triplicate, and values were expressed as mean ± standard deviation (SD).

**Western blotting**

Protein lysates were extracted using cell lysis buffer containing a protease inhibitor cocktail (Roche Diagnostics GmbH, Mannheim, Germany). Subsequently, protein samples were separated by SDS-PAGE, and electro-transferred to PVDF membranes. Thereafter, immunoblots were probed with the indicated primary antibodies (Table S2). Finally, protein bands were visualized using Clarity™ Western ECL Substrate (Bio-Rad Laboratories).

**Immunohistological staining**

Tissue sections from paraffin-embedded human or mouse tumors were incubated overnight with the indicated primary antibodies. Subsequently, the slides were incubated with a secondary anti-mouse or anti-rabbit IgG antibody (ZSGB-BIO, Beijing, China) and visualized using 3,3'-diaminobenzidine (DAB, ZSGB-BIO, ZLI-9019). Stained slides were scanned using a Pannoramic Scan 250 Flash or MIDI system, and images were acquired using Pannoramic Viewer 1.15.2 (3DHISTECH Kft., Budapest, Hungry). The quantitative score of the tissue sections was calculated according to the staining intensity and percentage of positive cells. Staining intensity was scored from 0 to 3 (0, negative; 1, weak; 2, moderate; and 3, strong). The H-score was obtained using the following formula: 1 × *X*1 + 2 × *X*2 + 3 × *X*3 with a range of 0–300, where *X* indicates the percentage of positively stained cells: *X*1 indicates weak staining, *X*2 indicates moderate staining, and *X*3 strong staining.

**PTBP3 expression and purification**

His-tagged pET28a-PTBP3-RRM3/4 expressed in *Escherichia coli* BL21 (DE3) was induced using 0.5 mM isopropyl β-D-1-thiogalactopyranoside (IPTG). Next, cells were resuspended in lysis buffer containing 20 mM Tris-HCl (pH 8.0), 300 mM NaCl, and 5% glycerol, followed by sonication for 40 min. The lysates were then puriﬁed using a Ni-NTA afﬁnity column (GE Healthcare, Chicago, IL, USA). Finally, proteins were eluted with elution buffer.

**Dual immunofluorescence staining**

Huh7 or SNU-449 cells were fixed with frozen methanol and blocked with 5% BSA for 1 h. Subsequently, the cells were incubated at 4 °C overnight with rabbit anti-E-cadherin (Abcam, Cambridge, UK) and mouse anti-vimentin (Santa Cruz Biotechnology Inc., Dallas, TX, USA) primary antibodies, followed by incubation with Alexa Fluor 488 or 594 secondary antibody (Invitrogen). Nuclear staining was performed using DAPI (10236276001; Roche Diagnostics GmbH). Sections were then examined using a laser scanning confocal microscope (Leica TCS SP8, Leica Microsystems, Wetzlar, Germany).

**Drug Affinity Responsive Target Stability**

Drug affinity responsive target stability (DARTS) was conducted to identify the potential targets of UDP-GlcUA. Briefly, 3 × 10^7^ cells were lysed in M-PER (#78501, Thermo Fisher Scientific) with a protease inhibitor cocktail and phosphatase inhibitor cocktail. TNC buffer (50 mM Tris-HCL pH 8.0, 50 mM NaCl, and 10 mM CaCl_2_) was then added to the lysate. Subsequently, cell lysates at 4 µg/µL were incubated with varying concentration of UDP-GlcUA or PBS for 1 hr at room temperature and then digested with [Pronase](https://www.sciencedirect.com/topics/biochemistry-genetics-and-molecular-biology/pronase) (1:1500 for PTBP3) for 30 min at room temperature. The digestion was stopped by adding a protease inhibitor cocktail, and the samples were immediately boiled with 2 × loading buffer for western blotting. GAPDH was used as a negative control.

**Cellular Thermal Shift Assay**

Cellular thermal shift assay (CETSA) was performed to investigate the thermal stability changes of PTBP3 protein in *cellular*. Briefly, 1 × 10^7^ Huh7 cells were pretreated with 500 μM UDP-GlcUA for 2 hrs at room temperature. Subsequently, cells were suspended with PBS containing a protease inhibitor cocktail, and subjected to five [freeze-thaw](https://www.sciencedirect.com/topics/biochemistry-genetics-and-molecular-biology/freeze-thawing) cycles to [lyse cells](https://www.sciencedirect.com/topics/biochemistry-genetics-and-molecular-biology/cytolysis). Then, equal volumes of protein supernatant were heat shocked in an eppendorf thermal cycler at the indicated temperature for 3 min to denature proteins, and immediately cooled down at room temperature for 3 min. Finally, the supernatant was boiled with 2 × loading buffer for [western blotting](https://www.sciencedirect.com/topics/biochemistry-genetics-and-molecular-biology/western-blot). The bands were quantified using the Image-Pro Plus analyzer software and plotted with three biological replicates.

**Transwell migration assays**

Huh7 (4 × 10 ^4^), SK-Hep1 (2 × 10 ^4^) or SNU-449 (3 × 10 ^4^) cells were seeded onto the upper chamber of a Transwell filter (8.0 μm pores, Falcon, USA) with serum-free medium. After 24 h, cells were fixed with 4% paraformaldehyde in PBS. Non-migrated cells on the upper chamber were removed with a cotton swab, and the migrated cells on the underside of the filter were stained with crystal violet staining solution (Beyotime Biotechnology). In parallel, cells were also separately plated onto plates without Transwell filters to determine the total number of attached cells. For each experiment, the number of migrated cells in five random fields was counted, and three independent experiments were performed.

**Wound healing assays**

Cells proliferated in standard culture condition until confluent, followed by scratching on cell monolayer using Wound Maker (Essen Bioscience, Ann Arbor, MI, USA). After wounding, cells were washed once with growth medium and further incubated with serum-reduced medium for 48 h. During this period, real-time gap distances were imaged using an IncuCyte ZOOM Live-Cell Imaging system (Essen Bioscience).

**Reference**

1. Luo J, Deng Z, Luo X*, et al.* A protocol for rapid generation of recombinant adenoviruses using the AdEasy system. *Nature protocols* 2007; **2**: 1236-1247.

2. Yang F, Li J, Deng H*, et al.* GSTZ1-1 Deficiency Activates NRF2/IGF1R Axis in HCC via Accumulation of Oncometabolite Succinylacetone. *The EMBO journal* 2019; **38**: e101964.

3. Gao Q, Zhang G, Zheng Y*, et al.* SLC27A5 deficiency activates NRF2/TXNRD1 pathway by increased lipid peroxidation in HCC. *Cell death and differentiation* 2020; **27**: 1086-1104.

4. Sánchez-Rivera F, Papagiannakopoulos T, Romero R*, et al.* Rapid modelling of cooperating genetic events in cancer through somatic genome editing. *Nature* 2014; **516**: 428-431.

**Figures and Figure legends**


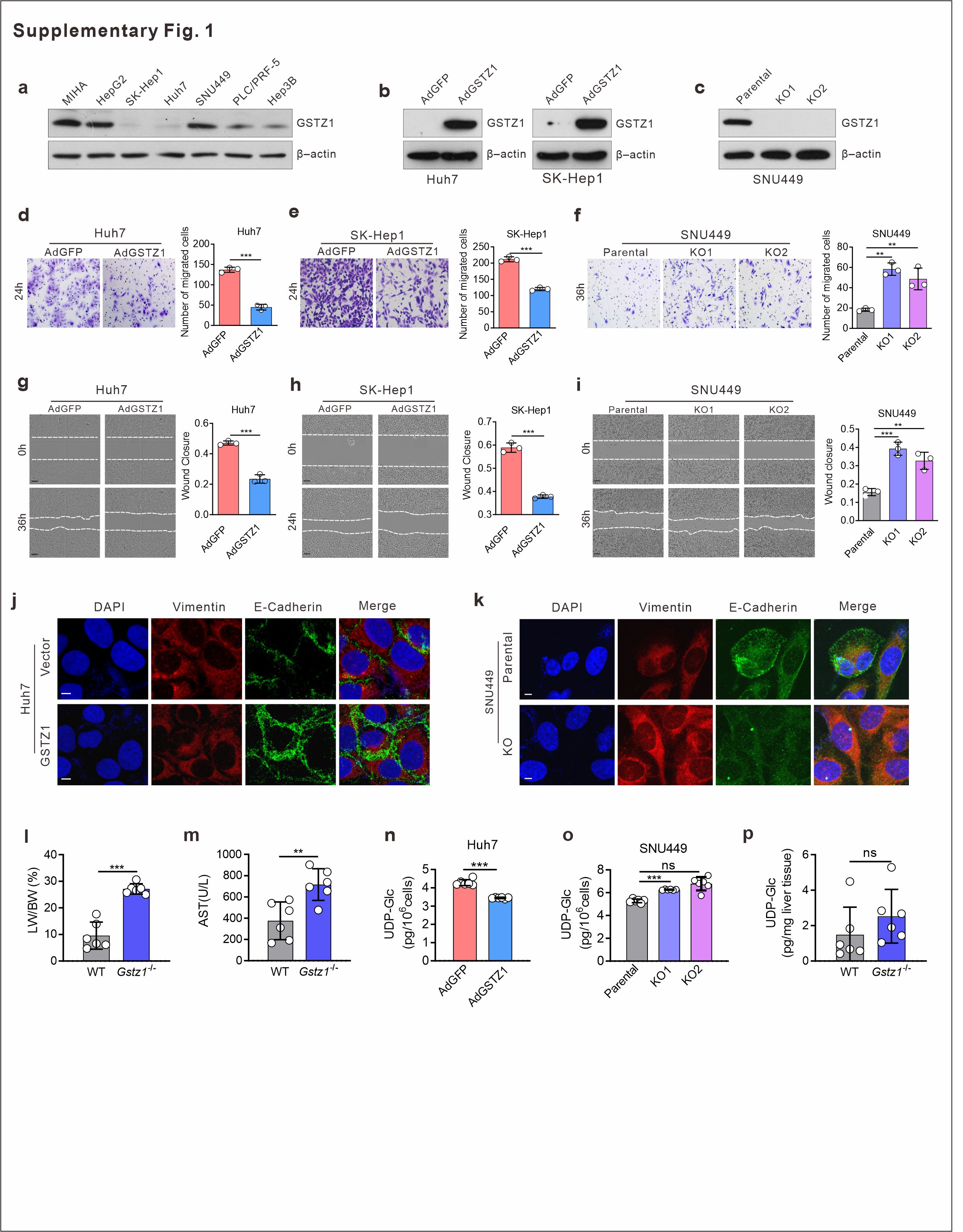


**Supplementary Fig. 1. GSTZ1 inhibits hepatocellular carcinoma (HCC) metastasis and glucuronate pathway activity**. (a) The endogenous expression levels of GSTZ1 protein in hepatoma cell lines. (b) Overexpression of GSTZ1 in Huh7 and SK-Hep1 cells was confirmed by immunoblot assay. (c) GSTZ1 knockout (KO) SNU449 cells were established by CRISPR-Cas9 system, the knockout efficiency was confirmed by Western blot analysis. (d–f) Representative images of transwell migration assays and quantification of the migrated cells in Huh7 (d) or SK-Hep1 (e) cells infected with AdGSTZ1 or AdGFP, or GSTZ1-KO SNU449 cells (f) (*n* = 3 independent experiments). (g–i) Representative images of wound healing assays and quantification of wound closure in Huh7 (g) or SK-Hep1 (h) cells infected with AdGSTZ1 or AdGFP, or GSTZ1-KO SNU449 cells (i) (*n* = 3 independent experiments). Scale bar: 200 μm. (j–k) Representative immunofluorescence staining of E-cadherin and vimentin from three independent experiments. Scale bar: 100 μm. (l–m) Quantification of liver-to-body weight (LW/BW) ratios and serum aspartate aminotransferase (AST) activity in WT and *Gstz1*^-/-^ mice (*n* = 6). (n–p) UDP-Glc levels in GSTZ1-OE Huh7 cells (n), GSTZ1-KO SNU449 cells (o), and *Gstz1*^-/-^ mice liver tissues (p). Data are mean ± SD. *P-* values were derived from an unpaired, two-tailed Student’s *t-*test in (d–e, g–h, and n); Mann-Whitney U test in (l–m, p); one-way ANOVA followed by the Tukey test in (f, i, and o). (** *p* < 0.01, ****p*< 0.001).


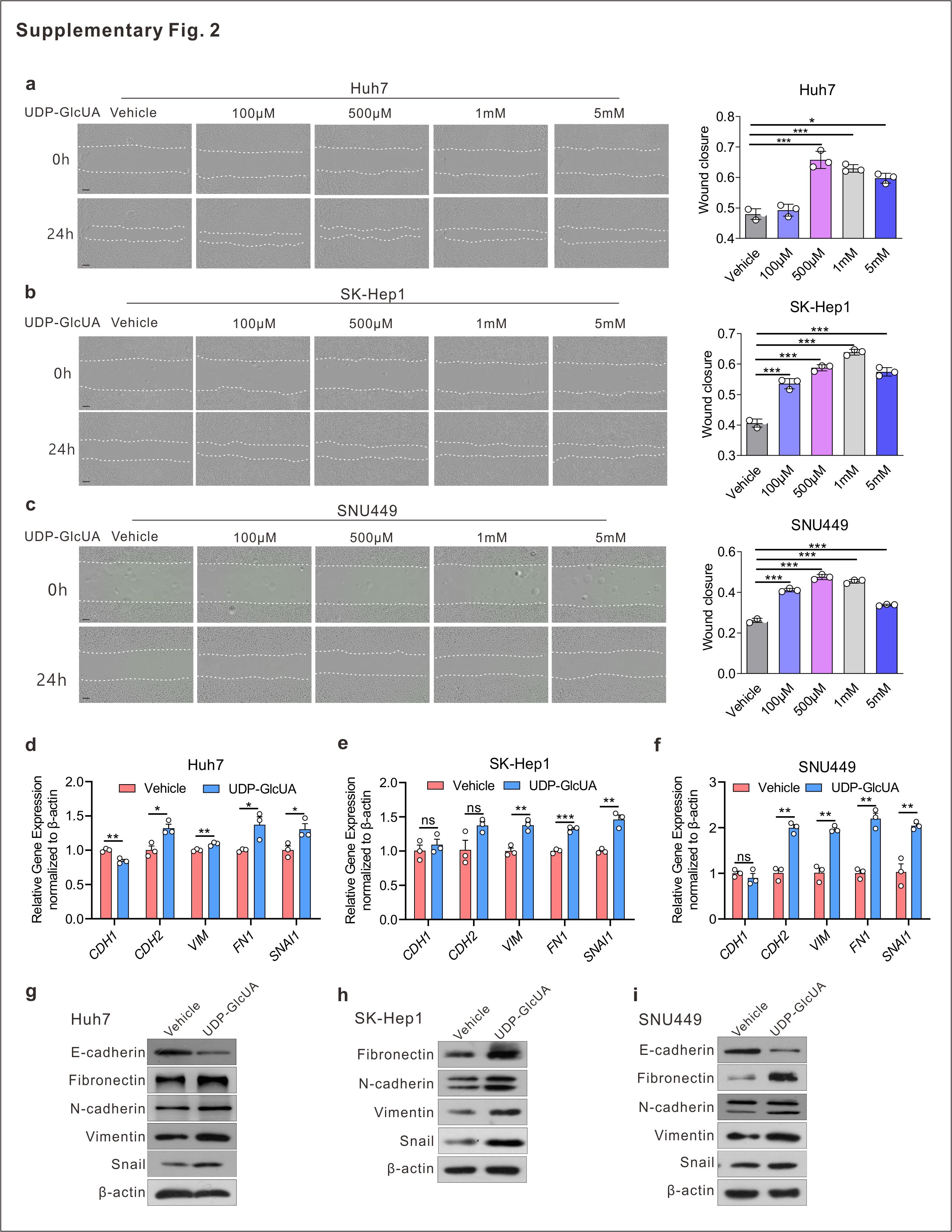


**Supplementary Fig. 2. UDP-GlcUA promotes hepatoma cell migration and epithelial-mesenchymal transitions**. (a–c) Representative images of wound healing assays and quantification of wound closure in Huh7 (a), SK-Hep1 (b), and SNU449 (c) cells supplemented with different doses of UDP-GlcUA (*n* = 3 independent experiments). Scale bar: 200 μm. (d–f) qRT-PCR analysis of epithelial-to-mesenchymal transition (EMT)-related genes *CDH1*, *CDH2*, *VIM*, *FN1*, and *SNAI1* (*n* = 3). (g–i) Immunoblots of EMT-related proteins. Data are mean ± SD. *P*-values were derived from an unpaired, two-tailed Student’s *t*-test in (a–c), and (d–f). (* *p* < 0.05, ** *p* < 0.01, ****p* < 0.001).


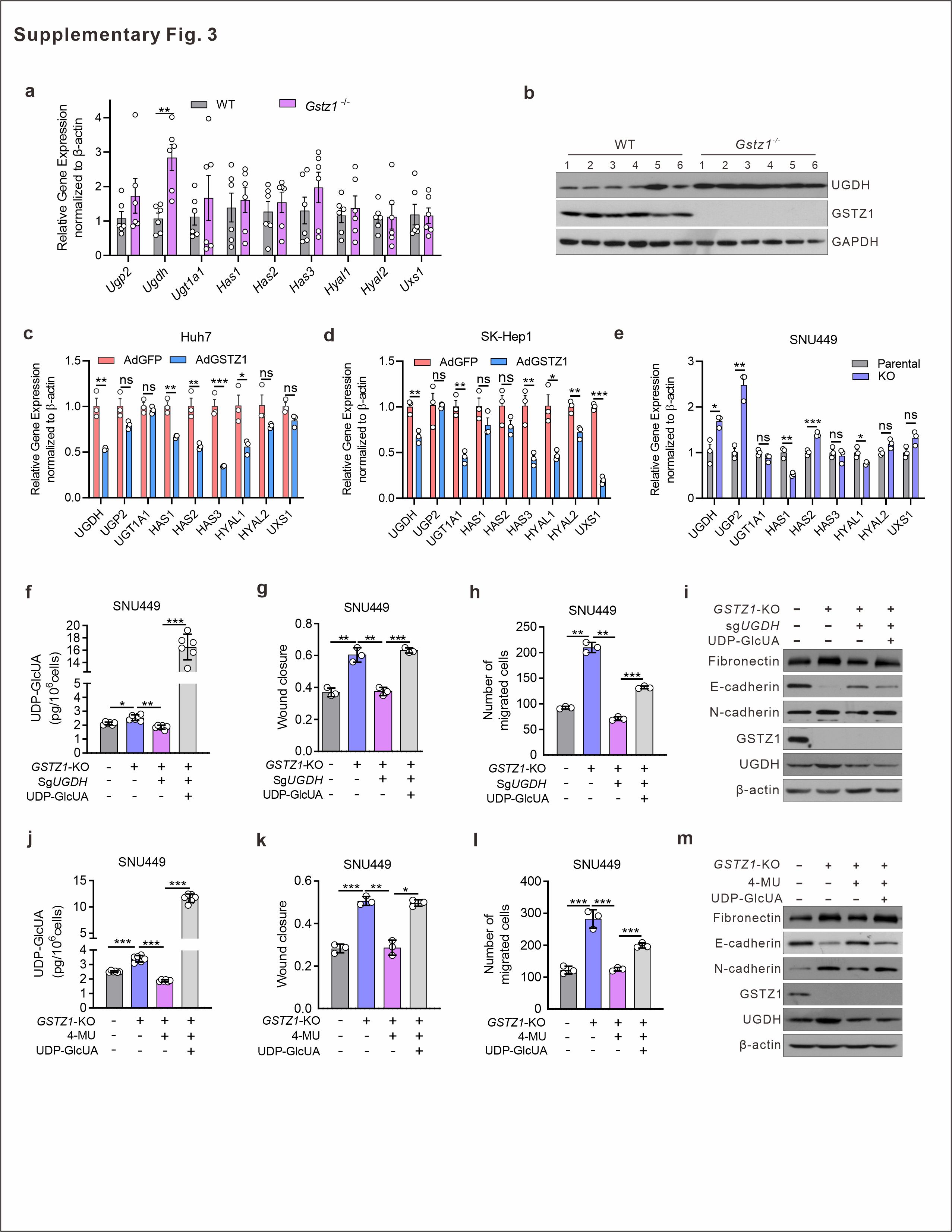


**Supplementary Fig.3. UGDH-mediated UDP-GlcUA accumulation promotes hepatoma cell migration upon GSTZ1 loss**. (a-b) qPCR for indicated genes and immunoblotting for indicated proteins in wild-type (WT) (*n* = 6) and *Gstz1*^-/-^ (*n* = 6) mice liver tissues. (c–e) qPCR of glucuronic pathway-related genes in Huh7 (c) or SK-Hep1 (d) cells infected with AdGSTZ1 or AdGFP, or GSTZ1-KO SNU449 cells (e). (f–i) UDP-GlcUA levels (*n* = 6) (f), quantification of wound closure (g), migrated cells (h), and immunoblots of epithelial-to-mesenchymal transition (EMT)-related proteins (i) in *GSTZ1*-KO SNU-449 cells with UGDH depletion after 0.5 h of UDP-GlcUA treatment. *n* = 3 independent experiments. (j–m) UDP-GlcUA levels (*n* = 6) (j), quantification of wound closure (k) and migrated cells (l), and immunoblots of epithelial-to-mesenchymal transition (EMT)-related proteins (m) in *GSTZ1*-KO SNU-449 cells supplemented with 4-MU (500 μM, 24 h) and UDP-GlcUA (500 μM, 0.5 h). Data are mean ± SD. *P*-values were derived from an unpaired, two-tailed Student’s *t*-test in (a, c–e); one-way ANOVA followed by the Tukey test in (f–h) and (j–l) (* *p* < 0.05, ** *p* < 0.01, ****p* < 0.001).


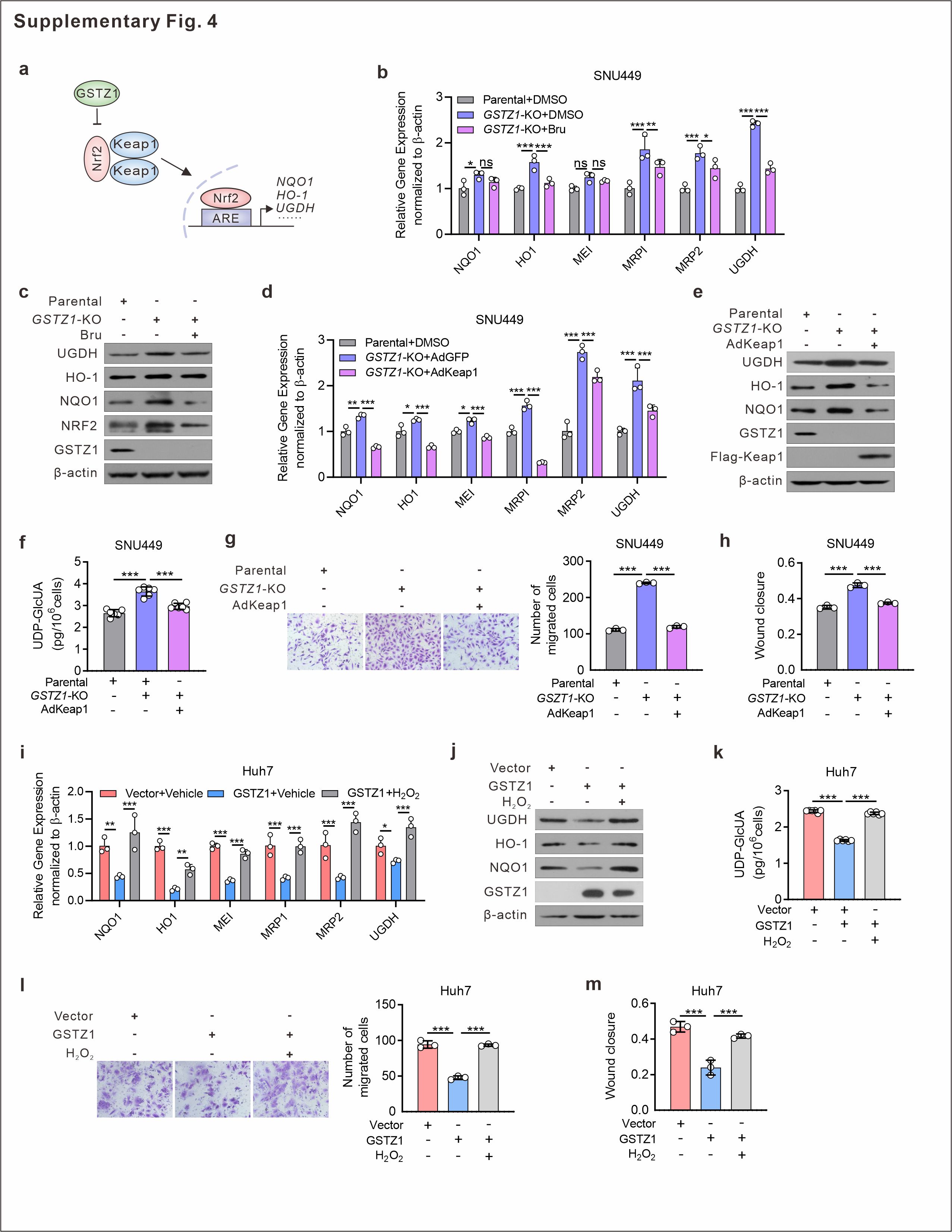


**Supplementary Fig.4. GSTZ1 deficiency-mediated glucuronate pathway activation are NRF2 dependent.** (a) Proposed activation model of the Keap1/NRF2 pathway in GSTZ1-deficient hepatocellular carcinoma (HCC). (b–e) qPCR for indicated genes and immunoblots of NRF2 downstream proteins in GSTZ1-KO SNU-449 cells supplemented with Bru (60 nM, 24 h) (b, c) or infected with AdKEAP1 for 48h (d, e). (f-h) UDP-GlcUA levels (*n* = 6) (f), quantification of migrated cells (g) and wound healing assays (h) in GSTZ1-KO SNU-449 cells infected with AdKEAP1. Scale bar: 200 μm. (i–j) qPCR for indicated genes and immunoblots of NRF2 downstream proteins in GSTZ1-OE Huh7 cells supplemented with H_2_O_2_ (100 μM) for 6 h. (k–m) UDP-GlcUA levels (*n* = 6) (k), quantification of migrated cells (l), and wound healing assays (m) in GSTZ1-OE Huh7 cells supplemented with H_2_O_2_ (100 μM) for 6 h. Scale bar: 200 μm. *n* = 3 independent experiments. Data are mean ± SD. *P*-values were derived from one-way ANOVA followed by the Tukey test in (b, d, g–h, and k–m) (* *p* < 0.05, ** *p* < 0.01, ****p* < 0.001).


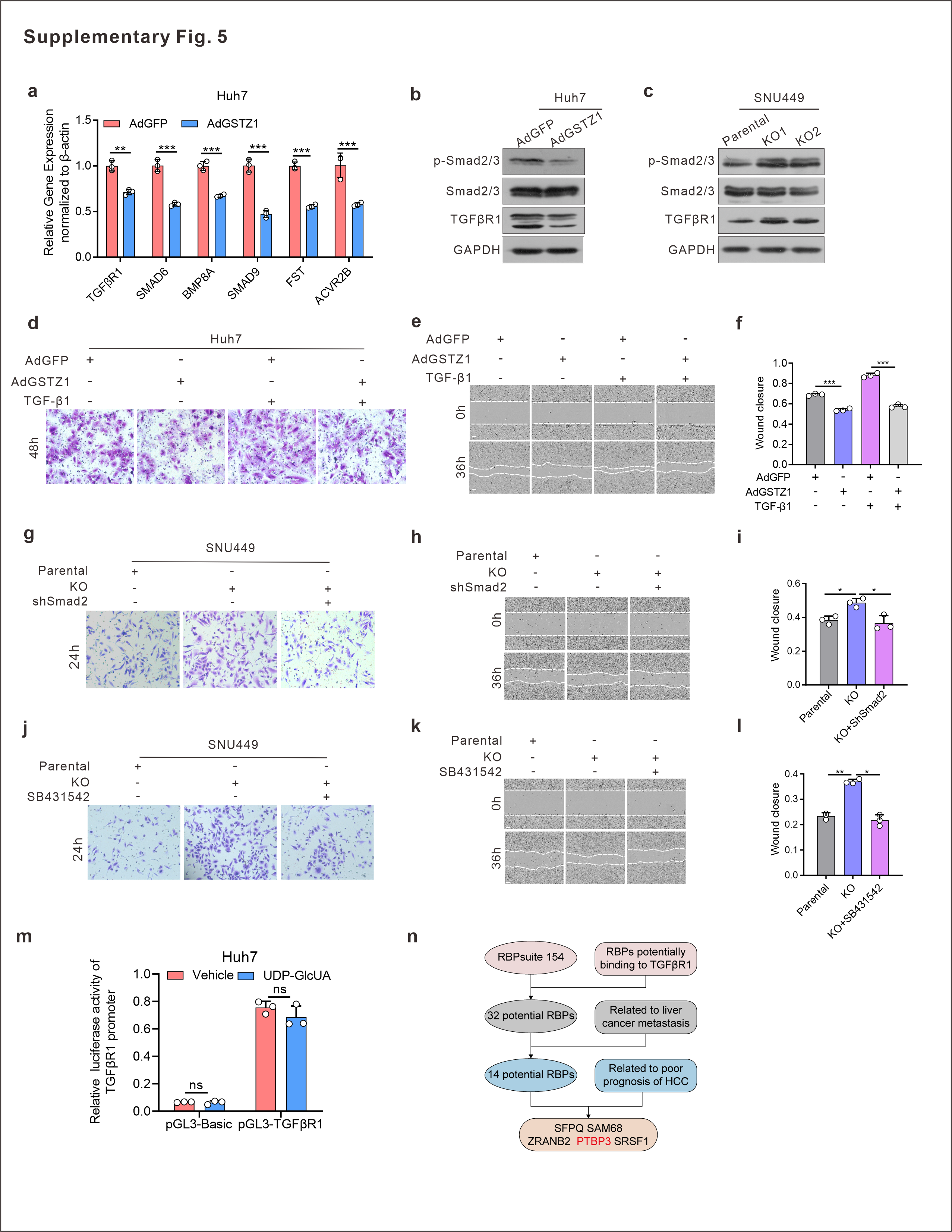


**Supplementary Fig. 5. GSTZ1 inhibits transforming growth factor-β/Smad signaling.** (a) qPCR of TGFβ/Smad pathway-related genes in Huh7 cells infected with AdGSTZ1 or AdGFP (*n* = 3, each). (b–c) Immunoblots of pSmad2/3, and Smad2/3 in GSTZ1-OE Huh7 cells (b) or GSTZ1-KO SNU449 cells (c). (d–l) Transwell migration assays, wound healing assays and quantification of wound closure in GSTZ1-OE Huh7 cells supplemented with or without TGFβ1 (10 ng/ml, 24h) (d–f), GSTZ1-KO SNU-449 cells transfected with shSmad2 (g–i) or supplemented with SB431542 (10 μM, 36h) (j–l). Scale bar: 200 μm. (m) Relative transcriptional activities in Huh7 cells as assessed by TGFβR1-luciferase assays (*n* = 3 independent experiments). (n) Workflow analysis of potential RNA-binding proteins (RBPs) binding to *TGFβR1* mRNA. Data are mean ± SD. *P*-values were derived from an unpaired, two-tailed Student’s *t*-test in (a and m) and one-way ANOVA followed by the Tukey test in (f, i, and l) (* *p* < 0.05, ** *p* < 0.01, ****p* < 0.001).


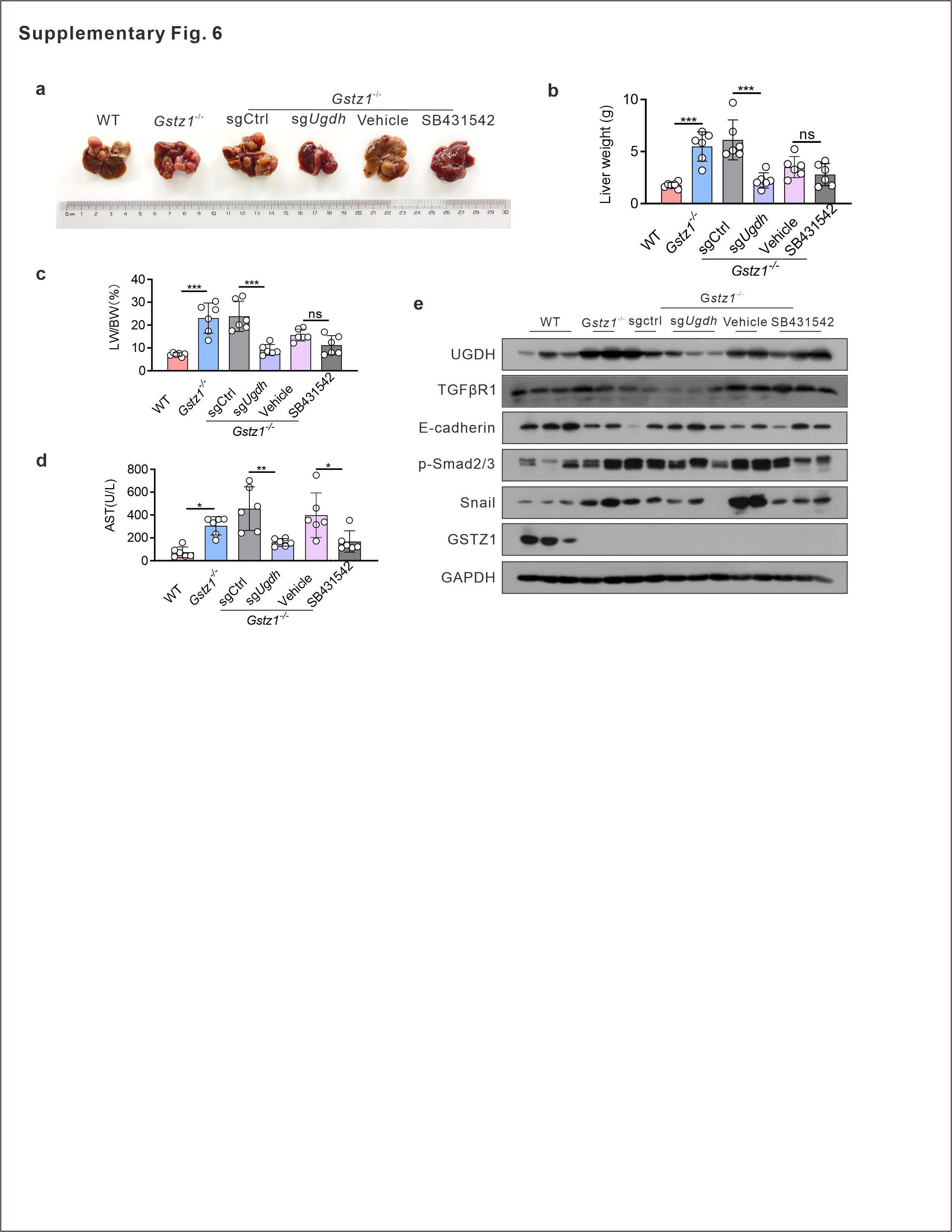


**Supplementary Fig. 6. Blockage of the glucuronic pathway or TGFβ signaling blunts hepatocellular carcinoma (HCC) metastasis driven by Gstz1 loss.** (a) Liver gross appearance of WT and *Gstz1^-^*^/-^ mice. (b–d) Quantification of Liver weight, liver-to-body weight (LW/BW) ratios and serum aspartate aminotransferase (AST) activity (*n* = 6). (e) Immunoblots of UGDH, TGFβR1, E-cadherin, pSmad2/3, Snail, and GSTZ1 proteins. Data are mean ± SD. *P*-values were derived from one-way ANOVA followed by the Tukey test in (b–d) (* *p* < 0.01, ** *p* < 0.01, ****p* < 0.001).

| **Table S1. Primer sequences used in this study** | | | |
| --- | --- | --- | --- |
| **Primers for real-time PCR Sense Primer (5'-3') Antisense Primer (5'-3')** | | | |
| SNAI1 | AAGATGCACATCCGAAGCCA | | ATCTGAGTGGGTCTGGAGGT |
| VIM | GAGAACTTTGCCGTTGAAGC | | TCCAGCAGCTTCCTGTAGGT |
| CDH2 | CCGAGCCCCAGTATCCG | | GAAGTTCAGTCATCACCTCCACC |
| FN1 | AGGCTTGAACCAACCTACGG | | CTCCCTGACGGTCCCACTT |
| VTN | CCAAGAAACAAAGGTTTAGGCA | | CAGGTGGCAGGCACAAGC |
| CRB3 | GAGAAGCGGCAGACGGAG | | TGCACAGAGCCCACCACA |
| OCLN | CGAAGAAAGATGGACAGGTATGA | | CCACCGCTGCTGTAACGAG |
| UGP2 | AAGAAGTCATTCGGCAAGAGC | | CCACCATTGAGTTTCACCACC |
| UGDH | GCTCGTGACCATTTCCAAGG | | TTCGGAATTTCACCAGAAGGA |
| UGT1A1 | CCAAAATCCACTATCCCAGGA | | GGGTCATCGGGTGACCAA |
| HAS1 | CTCGGAGATTCGGTGGACTA | | GAAACTGCTGCAAGAGGTTATTC |
| HAS2 | GGGGCACATCAGGAAGGA | | TCAAAAGCATGACCCAATAGC |
| HAS3 | GTGGCTCAACCAGCAAACC | | CGTCAGCAGGAAGAGGAGAATG |
| HYAL1 | GCCCTCTTCCTGACCTTACTC | | CCTGGGTTGGCTACCACATC |
| HYAL2 | CGCTGCCCTGATGTTGAG | | CGGCTGTAGGTGGGTCGT |
| UXS1 | AGCCTCCCCTCCAAACTACA | | GGCCACTCGCACTTCCAC |
| CDH1 | AATTGCTCACATTTCCCAACTC | | CTCTGTCACCTTCAGCCATCC |
| NQO1 | CCAGCAGACGCCCGAAT | | CCAAGTGATGGCCCACAGA |
| ME1 | AGGGCATATTGCTTCAGTTCTC | | TTTCGGTTCCCACATCCAG |
| HO1 | AACTTTCAGAAGGGCCAGGT | | CTGGGCTCTCCTTGTTGC |
| MRP1 | ACCAAGTGCTTTCAGAAC | | AGAGATAGAGGAAGTAGAAGG |
| MRP2 | ACGGGCACATCACCATCAAG | | CTCCAGGCAGCATTTCCAAG |
| TGFβR1 | CCCATCAGTTGAAGAAATGAGAA | | CCTGTTGACTGAGTTGCGATAA |
| BMP8A | GGAGTTCCGCTTTGACCTGA | | CTCGGAGCGTCTGAAGATCC |
| SMAD9 | ACGCCACCTATCCTGACTCT | | CAGAGGCTTCTGTGGCATGA |
| FST | TGTGAGAACGTGGACTGTGG | | ACTTCCAGTTCTGGCTGCTC |
| ACVR2B | GAGACGGTACATGGCTCCTG | | GTGCTGGCCAATCTCTTCCT |
| SMAD2 | GCCACGGTAGAAATGACAAGAA | | TTAACAGACTGAGCCAGAAGAGC |
| SMAD3 | CCCAACTGTAACCAGCGCTA | | CTTTGACGAAGCTCATGCGG |
| SMAD4 | GCCATCTTCAGCACCACCC | | CAAACAAAAGCGATCTCCTCC |
| SMAD7 | GTGCGTGGTGGCATACTGG | | GGGTAACTGCTGCGGTTGTAC |
| Ugp2 | GGAGCTGCAATTAAAAGTTTCG | | ACCAAGGGCACTGTAGGGA |
| Ugdh | CTTTGATGGTCGGCGTGTC | | TGGGTTTCTTGTTAGGTGGGT |
| Ugt1a1 | ACGCTGGGAGGCTGTTAGT | | AAAGGCAGTCCGTCCAAGTT |
| Has1 | GGCTGTTGGAGGGGATGT | | GGACCACTGATGCAGGACAC |
| Has2 | TGAACAAAACGGTAGCACTCTG | | GTCCCTGCCCATAACTTCG |
| Has3 | CCTGGGTCTACATCTGCTCATC | | GTATTCGGGGTCCTCTTGGT |
| Hyal1 | CTGGGACAGCAAGGACATTT | | GTCAGGGAAGCCATAGTAGCC |
| Hyal2 | TGGGGCGACTCGGAAGA | | GCATTGAGGTGCAGGAAGG |
| Uxs1 | ACCCCGAGCCTGCTATGA | | TGGAATGCCCGTGTCTGA |
| **Primers for sub-clone (TGFβR1 3' UTR for PTBP3 binding)** | | | |
| pGL3-Basic-  WT | | CTAGCTAGCTCATTGAAGCAAATGAATGA | CTAGCTAGCGATGGGCAGTATAAACAACTGT |
| pGL3-Basic-  Mut 1 | | TGTGATCAGGTACTTATATAGTGGGGGTTTTTT | AACCCCCACTATATAAGTACCTGATCACAGAAT |
| pGL3-Basic-  Mut 2 | | GTTAATTCCTTATATGGCTAGGGATGGTTTGA | AACCATCCCTAGCCATATAAGGAATTAACTGC |
| **Primers for sub-clone** | | | |
| pET28a-His- PTBP3 (1-552aa) | | TGCGGATCCATGGATGGTGTTGTTACAGATC | CCCAAGCTTTCAGATTGTAGATTTTGAGAAGGA |
| pET28a-His- PTBP3-RRM3/4  (357-552aa) | | TGCGGATCCAATTCTGTTCTACTCGTCACAAAT | CCCAAGCTTTCAGATTGTAGATTTTGAGAAGGA |
| **Primers for sgRNA** | | | |
| GSTZ1-sgRNA | CACCGCACGCTGGCCCTCTTCTTT | | AAACAAAGAAGAGGGCCAGCGTGC |
| GSTZ1-sgRNA | CACCGCCCAGAACGCCATCACTTG | | AAACCAAGTGATGGCGTTCTGGGC |
| UGDH-sgRNA | CACCGGTAGCAACAGCGATTGGAA | | AAACTTCCAATCGCTGTTGCTACC |
| Ugdh-sgRNA | CACCGAAGAGGTGGCAACGGCCAT | | AAACATGGCCGTTGCCACCTCTTC |
| PTBP3- sgRNA | CACCGGCCAGGCTTCCTGATTGGA | | AAACTCCAATCAGGAAGCCTGGCC |
| lentiCRISPR-v2 | GGTTTATTACAGGGACAGCAG | | ACACGACATCACTTTCCCAG |

**Table S2. Antibodies used for Western blotting**

**(WB), IP, IHC and IF.**

| **Antibodies** | **Source** | **Antibody dilution** | **Cat no.** |
| --- | --- | --- | --- |
| Rabbit anti GSTZ1 | GeneTex, USA | 1:1000 for WB; 1:200 for IHC | GTX106109 |
| Rabbit anti-UGDH | GeneTex, USA | 1:2000 for WB; 1:500 for IHC | GTX104993 |
| Rabbit anti-HO-1 | GeneTex, USA | 1:1000 for WB | GTX101147 |
| Goat anti-rabbit,  secondary | Abcam, USA | 1:5000 for WB | ab6721 |
| Goat anti-mouse,  secondary | Abcam, USA | 1:10000 for WB | ab6789 |
| Rabbit anti-Snail/Slug | Abcam, USA | 1:1000 for WB; 1:200 for IHC | ab180714 |
| Rabbit anti-E-cadherin | Abcam, USA | 1:1000 for WB; 1:200 for IF | ab40772 |
| Mouse anti-Ecadherin | Abcam, USA | 1:200 for IHC | ab231303 |
| Rabbit anti-Smad2/3 | Cell Signaling Technology, USA | 1:1000 for WB; 1:200 for IF | 8685 |
| Rabbit anti-pSmad2/3 | Cell Signaling Technology, USA | 1:1000 for WB; 1:200 for IHC | 8828 |
| Rabbit anti-N-cadherin | Cell Signaling Technology, USA | 1:1000 for WB | 13116 |
| Mouse anti-Fibronectin | Santa cruz Technology, USA | 1:500 for WB | sc59826 |
| Mouse anti-Vimentin | Santa cruz Technology, USA | 1:2000 for WB; 1:200 for IF | sc6260 |
| Mouse anti-GAPDH | Beyotime Biotechnology, China | 1:3000 for WB | AG019 |
| Mouse anti-β-Tublin | Proteintech, USA | 1:5000 for WB | 66240-1-Ig |
| Rabbit anti-PTBP3 | Proteintech, USA | 1:1000 for WB; 1:200 for IP | 14027-1-AP |
| Rabbit anti-β-actin | Biosharp, China | 1:3000 for WB | BL005B |
| Rabbit anti-NQO1 | Bioworld, USA | 1:1000 for WB | BS6833 |
| Rabbit anti-Lamin B1 | Bioworld, USA | 1:5000 for WB | AP6001 |
| Mouse anti-His | Thermo Fisher Scientific, USA | 1:1000 for WB | MA1-21315 |
| Goat anti-Rabbit IgG,  Alexa Fluor ® 488 | Invitrogen, USA | 1:500 for IF | A-11034 |
| Goat anti-Mouse IgG, Alexa Fluor ®594 | Invitrogen, USA | 1:500 for IF | A-21125 |
| Rabbit anti-TGFβR1 | BOSTER, China | 1:1000 for WB; 1:100 for IHC | BA0294 |
| Mouse anti-Flag | Sigma-Aldrich, USA | 1:1000 for WB | F3165 |

| **Table S3: Chemicals, Critical Commercial Assays and Experimental Models.** | | |
| --- | --- | --- |
|  |  |  |
| **Name** | **Source** | **Identifier** |
| **Chemicals** | | |
| Recombinant human TGFβ1 | Novoprotein Scientific Inc | G8769 |
| SB431542 | Selleckchem | S1067 |
| 4-Methylumbelliferone (4-MU) | MedChemExpress | HY-N0187 |
| Brusatol | Dalian Meilunbio Co. Ltd. | MB7292 |
| 5,6-Dichloro-1-beta-Ribo-furanosyl benzimidazole (DRB) | TargetMol | T7789 |
| Carbon tetrachloride (CCl_4_) | Macklin | C805332 |
| Diethylnitrosamine (DEN) | Sigma-Aldrich | N0756 |
| DMEM | HyClone | SH30243.01 |
| RPMI-1640 medium | HyClone | SH30027.01 |
| Opti-MEM | Gibco | 31985070 |
| Penicillin-Streptomycin Solution | HyClone | SV30010 |
| Trypsin | Gibco | 15050057 |
| Trypsin-EDTA | Gibco | 25200072 |
| cOmplete™, EDTA-free Protease Inhibitor Cocktail Tablets | Roche Diagnostics GmbH | 04693132001 |
| Cell lysis buffer for Western | Beyotime Biotechnology | P0013 |
| DAPI | Roche Diagnostics GmbH | 10236276001 |
| TRIzol™ Reagent | Invitrogen | 15596026 |
| iTaq™ Universal SYBR® Green Supermix | Bio-Rad | 1725121 |
| 2×Taq PCR Green Mix | Dinguo | PER 007 |
| Matrigel basement membrane matrix | Corning | # 354234 |
| Pierce™ streptavidin agarose beads | Thermo Fisher Scientific | # 20347 |
|  | | |
| **Critical Commercial Assays** | | |
| Magna RIP™ RNA-Binding Protein Immunoprecipitation Kit | Millipore | # 17-701 |
| Nuclear and Cytoplasmic Protein Extraction Kit | Beyotime Biotechnology | P0027 |
| DAB kit | ZSGB-BIO | ZLI-9019 |
|  | | |
| **Experimental Models** | | |
| BALB/c nude mice (male) | Experimental Animal Center of Chongqing Medical University | N/A |
| *Gstz1^-/-^ C57 BL/6J mice* | Lab stock | N/A |

**Table S4: 154 RBPs through eCLIP-seq from ENCODE corresponding to human genome hg19 version.**

| AARS | CSTF2T | EIF3G | GNL3 | ILF3 | NONO | PUS1 | SF3B1 |
| --- | --- | --- | --- | --- | --- | --- | --- |
| EIF3H | GPKOW | KHDRBS1 | NPM1 | QKI | SF3B4 | TBRG4 | XRCC6 |
| KHSRP | NSUN2 | RBFOX2 | SFPQ | TIA1 | XRN2 | AGGF1 | DDX3X |
| RBM15 | SLBP | TIAL1 | YBX3 | AKAP1 | DDX42 | EXOSC5 | GTF2F1 |
| TNRC6A | YWHAG | AKAP8L | DDX51 | FAM120A | HLTF | LIN28B | PCBP1 |
| APOBEC3C | DDX52 | FASTKD2 | HNRNPA1 | LSM11 | PCBP2 | RBM5 | SND1 |
| FKBP4 | HNRNPC | MATR3 | PHF6 | RPS11 | SRSF1 | U2AF1 | ZNF622 |
| METAP2 | POLR2G | RPS3 | SRSF7 | U2AF2 | ZNF800 | BCCIP | DDX6 |
| RPS5 | SRSF9 | UCHL5 | ZRANB2 | BCLAF1 | DGCR8 | FUBP3 | HNRNPM |
| UPF1 | BUD13 | DHX30 | FUS | HNRNPUL1 | NIP7 | PRPF4 | SAFB |
| FXR1 | HNRNPU | NIPBL | PRPF8 | SBDS | SUB1 | UTP3 | CPEB4 |
| PTBP1/2/3 | SDAD1 | SUGP2 | WDR3 | CPSF6 | EFTUD2 | G3BP1 | IGF2BP2 |
| WDR43 | CSTF2 | EIF3D | GEMIN5 | IGF2BP3 | NOLC1 | PUM2 | SF3A3 |
| AATF | EIF4G2 | LARP4 | RBM22 | TRA2A | AQR | FMR1 | MTPAP |
| DDX21 | GRSF1 | PABPC4 | SLTM | ZC3H11A | DDX55 | HNRNPK | PPIG |
| IGF2BP1 | SERBP1 | NKRF | SUPV3L1 | NCBP2 | STAU2 | DROSHA | NOL12 |
| TARDBP | ABCF1 | EWSR1 | LARP7 | RBM27 | TROVE2 | AUH | FTO |
| XPO5 | DDX24 | GRWD1 | PABPN1 | SMNDC1 | ZC3H8 | DDX59 | HNRNPL |
| PPIL4 | UTP18 | FXR2 | PUM1 | WRN | CDC40 | DKC1 | TAF15 |
| SAFB2 | SSB |  |  |  |  |  |  |
